# Burst statistics in an early biofilm quorum sensing model: the role of spatial colony-growth heterogeneity

**DOI:** 10.1038/s41598-019-48525-2

**Published:** 2019-08-19

**Authors:** Oliver Kindler, Otto Pulkkinen, Andrey G. Cherstvy, Ralf Metzler

**Affiliations:** 1 Institute for Physics & Astronomy, University of Potsdam, D-14476 Potsdam-Golm, Germany; 2 Institute for Molecular Medicine Finland and Helsinki Institute for Information Technology, University of Helsinki, FI-00014 Helsinki, Finland

**Keywords:** Computational biophysics, Statistical physics

## Abstract

Quorum-sensing bacteria in a growing colony of cells send out signalling molecules (so-called “autoinducers”) and themselves sense the autoinducer concentration in their vicinity. Once—due to increased local cell density inside a “cluster” of the growing colony—the concentration of autoinducers exceeds a threshold value, cells in this clusters get “induced” into a communal, multi-cell biofilm-forming mode in a cluster-wide burst event. We analyse quantitatively the influence of spatial disorder, the local heterogeneity of the spatial distribution of cells in the colony, and additional physical parameters such as the autoinducer signal range on the induction dynamics of the cell colony. Spatial inhomogeneity with higher local cell concentrations in clusters leads to earlier but more localised induction events, while homogeneous distributions lead to comparatively delayed but more concerted induction of the cell colony, and, thus, a behaviour close to the mean-field dynamics. We quantify the induction dynamics with quantifiers such as the time series of induction events and burst sizes, the grouping into induction families, and the mean autoinducer concentration levels. Consequences for different scenarios of biofilm growth are discussed, providing possible cues for biofilm control in both health care and biotechnology.

## Introduction

To ensure their evolutionary survival microorganisms have evolved to feature certain quite intricate behavioural and developmental mechanisms. Facing various challenges of their environment, these microbes must be able to compete but also to cooperate with each other. To effectively fulfil these tasks certain microorganisms are able to differentiate between alternative modes of cellular life, for instance, a maverick single-cell mode versus communal behaviour in a microbial biofilm^1–11^. One trait developed by this evolutionary demand is the ability to sense the density of other microorganisms (same or different species) in their vicinity. This ability is called *Quorum Sensing (QS)*^12–18^, the mechanism interconnected with biofilm formation. QS is a well-developed molecular biochemical communication method in a range of biological species, and it is particularly well studied for bacteria such as *Escherichia coli*, Salmonella, Pseudomonas, or *Aliivibrio fischeri*. However, similar mechanisms also appear to prevail in Archaea, some plants, as well as for social insects such as ants or bees.

**Figure 1:**
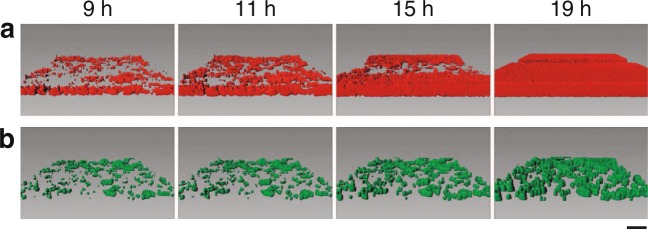
Snapshots of two evolving biofilms from^44^, the scale bar is 20 *μ*m. Depending on the growth conditions in these engineered biofilms (used for biorefinery purposes), different degrees of spatial cell heterogeneity are distinct in (**a**,**b**).

QS-bacteria produce specific molecules—low-molecular weight *autoinducers (AI)*—excreting them into the environment thereby establishing chemical gradients on the microscale. Within a given “signal range” (see below) bacteria sense the local density of AI molecules which is a function of the local density of bacteria, the overall cell positioning/clustering, and the AI diffusive properties. Once the AI density crosses a threshold value—corresponding to a sufficiently large local bacteria population density—a synchronisation between the bacteria is triggered, leading to social phenomena such as biofilm formation or bioluminescence^19–25^. In this QS process, the biochemical information from the surrounding is used to change the expression level of certain genes. Typically, the activation of genes at above-threshold AI levels leads to an up-regulation of the AI as well as other biomolecules stabilising the communal mode of the colony. In QS bacteria cells respond to extremely low concentrations of AIs: only a few molecules per cell are sufficient^26,27^, corresponding to nano-molar concentrations. For instance, the observed QS thresholds are 2.5 nM^28^ and 5 nM^29^ so that a few molecules can trigger induction. The ability of bacteria to finely sense changes in their local environments and to monitor gradients of nutrients, chemical signals, and oxygen concentration is a key prerequisite for the gene-regulation-based adaptation mechanisms that ultimately ensures evolutionary advantages for the growth and survival strategies of certain bacterial strains/colonies. In Fig. 1 we show experimental results for two evolving biofilms with different degree of spatial spreading.

Apart from the formation of biofilms^16,30^ various other traits on the community level may also be regulated by QS^31^, such as extracellular digestion by secretion of enzymes^32^, bioluminescence^12,33^, virulency and proliferation inside living organisms^34,35^, microbial competence^36^ or antibiotic resistance^5,7,8,37^. QS may also play a role in the regulation of immune cell responses^38^. An intriguing topic is the very question of survival “in the microbial jungle” in which different species of bacteria aim at outcompeting each other, for instance, by secretion of anti-microbial toxins to kill or to impair vicinal competitor cells or otherwise disturb the accumulation of competitors^39^, leading to bacterial “rock-paper-scissors games”^40,41^.

In the following we discuss QS in the language of biofilm formation, which (apart from its attractiveness from a biophysics point of view) is also of significant societal relevance. In fact, up to 65% of microbial and 80% of all chronic infections are due to microbes residing in biofilms^42^. This is of particular severity as bacteria in biofilms are often less accessible to antibiotics and—inter alia, due to decreased metabolism of “dormant cells” as well as elevated mutation levels—feature a high level of antibiotic resistance that can drastically increase the mortality rate from diseases caused by these infections^43^. For a more detailed introduction into properties of QS—such as the interpretation of different signals or the connection to anti-bacterial chemotherapy—the reader is referred to the studies^18,22,23^. Biofilms may also be useful in modern biotechnology, for instance, in biorefineries^44^. Moreover, anti-virulence strategies based on interference of bacterial QS mechanisms are possible^45,46^. Improved control of biofilm growth and predictability of biofilm spreading dynamics is, therefore, of relevance for a number of health care and industrial purposes.

Below we study the influence of spatial disorder on the dynamics of the transition from single-cell to communal, biofilm-forming behaviour. Namely, how is this induction process influenced by the spatial distribution of cells in the growing biofilm, in particular, the spreading of the daughter cells after cell divisions due to bacterial motility or diffusion? While the typical cell-cell distance in a growing colony can be a decisive factor for the local AI concentration and, thus, for the timing of the biofilm-induction event (see the recent study^47^), we explore what happens when the local cell density varies statistically. Such a spatially disordered scenario is natural for a realistic growing colony in which randomly placed seed cells divide and cover an increasing area, leading to local regions with varying cell density (see Fig. 1). This emerging spatial inhomogeneity^14,48^ will lead to higher-density clusters, in which induction events occur long before the entire colony is induced. We refer also to^14^ for the discussion of various effects of cell density, mass-transfer properties of the environment, and spatial distribution of bacteria onto AI concentration and QS. This is in contrast to homogeneous scenarios^47^, in which a concerted induction occurs (a mean-field description). As the local concentration after a given number of cell divisions is lower in the homogeneous situation the induction occurs later (local versus global QS)^47^.

After detailing the setup, we quantify the anticipated influence of spatial disorder for a single evolving colony. A statistical evaluation of the observed characteristics then follows. Our conclusions and an outlook are presented in the end. Figure numbers starting with S refer to the supplementary figures.

## System and Numerical Methods

Certain bacteria are known to detect foreign microorganisms or interpret multiple signals via the mechanism of QS^49–51^. We here focus on the mechanism of detecting their own species with a single signalling molecule species called the AI. This name stems from the fact that the emitting cell senses its own signalling molecule, and the extracellular AI concentration feeds back on the cell’s gene-regulation cascades^14,48^.

We note that for such intra-species communication Gram-negative bacteria such as *Vibrio fischeri* or *Pseudomonas aeruginosa* produce species-specific molecules from the class of N-acyl homoserine lactones (AHL) as transmitters^20,52,53^. In total 56 different AHLs were identified so far^54^. The bacterial cells use specialised oligopeptide receptor types to measure the extracellular concentration of AHL molecules^15^. After secretion the AI molecules act as a passively diffusing signal spreading until they either degrade or bind to and are detected by a receptor. The AHL-driven regulatory QS circuits are mainly governed by the Luxl-type protein responsible for AHL synthase, and the Luxr-type protein involved in AHL-response regulation^52^. These proteins act as transcription factors regulating the expression of QS-related genes. Below a given extracellular concentration of AHL molecules the AI synthase is expressed at a lower, basal level. However, above a certain threshold, in a positive feedback loop the cell changes the expression level of the gene controlling the synthase of the AHL molecules. This process is called “induction” and is a direct response to the local cell concentration. Since every QS-responsive cell in a single-species environment is secreting and detecting these molecules, the concentration-increase effect can build up quickly when critical population densities are reached.

Biofilm growth typically starts on a surface, only at later stages biofilms grow into the third dimension. The classical experimental scenario is a biofilm grown in a Petri dish, with the vertical height of the solution much smaller than the horizontal extension (see also Fig. 1). We choose below a two-dimensional description; the formulation can be extended to the third dimension (on top of the growing colony). We use a protocol of simulations written in R-code. The physical parameters classifying our system is the typical cell radius *r*_*c*_ and the initial number *n*_*c*_(*t* = 0) of seed cells in the circular simulations area of radius *R*.

The cell division rate is *γ*, and—as a simple model of bacterial motility or diffusion—we place the two daughter cells such that one next-generation cell occupies the same position as the mother cell {*x*_old_, *y*_old_}, while (according to the first modelling scenario) the second cell is displaced by a fixed distance ${d}_{{\rm{new}}}=\sqrt{{({x}_{{\rm{new}}}-{x}_{{\rm{old}}})}^{2}+{({y}_{{\rm{new}}}-{y}_{{\rm{old}}})}^{2}}$ and at a random azimuthal angle in the plane. In the second modelling scenario, the positions of displaced daughter cells obey a Gaussian distribution with the standard deviation *σ*_str_ along *x* and *y* axes, namely,

1 $$P({x}_{{\rm{new}}})=\frac{1}{\sqrt{2\pi {\sigma }_{{\rm{str}}}^{2}}}\exp (-\frac{{({x}_{{\rm{new}}}-{x}_{{\rm{old}}})}^{2}}{2{\sigma }_{{\rm{str}}}^{2}}).$$

We checked that both cell-spreading scenarios yield qualitatively similar results. In the figures below we provide the values of *d*_new_ and *σ*_str_ for the situations when, respectively, the first or the second scenarios were implemented in the simulations. We note that this scenario is a first approximation: in reality the direction of cell placement depends on the cell population density and displacement separations should reflect bacterial mobilities. Note that a number of biofilm-forming bacteria move in reality using flagellar motion (and do not jump in space). Moreover, the emerging flows can cause dispersion and the motility-to-biofilm transition is known to exist^55^. As we here focus on the effects of geometric disorder, however, neglecting such dynamic effects appears to be in order.

AI molecules are produced with a basal production rate *φ*; after induction *φ* is enhanced by the ratio *η* such that induced cells produce AIs with rate *φ*(1 + *η*). The decay rate of AI molecules is *δ* and they diffuse with the diffusion coefficient *D*. The threshold concentration for induction of AI QS-signalling molecules is *β*_thr_.

We set the radius of the circular model cells to *r*_*c*_ = 2.5 *μ*m, the initial colony radius to *R* = 100 *μ*m (unless specified otherwise), and the initial cell density to *ρ* = 0.0025 cells per *μ*m^2^. The mean number of cells 〈*n*_*c*_〉 = *ρπR*^2^ ≈ 78.5 (or 〈*n*_*c*_〉 = *ρπR*^2^ ≈ 314 when *R* = 200 *μ*m) is chosen from a Poisson distribution (see below). The cell division rate is *γ* = 1/[60 min], the basal AI production rate in the model is *φ* = 1/sec, and the induction threshold is *β*_thr_ = 30 AI molecules per *μ*m^2^ on the surface (unless otherwise indicated). All these values are considered as fixed, biologically motivated parameters^19–26^. The variations we study reflect changes in the spatial disorder of the growing cell colony. Otherwise the parameter space would be too large for the current scope.

Different approaches in simulating the growth of the bacteria colony can be chosen, such as lattice-free or lattice-based, interacting or non-interacting, as well as individual- or density-based. In lattice-based approaches new cells can only occupy points of the lattice, while in lattice-free approaches the whole plane is available for cell placement. A detailed comparison between the two approaches is reported in^56^. We choose the more natural lattice-free strategy. We follow an individual-based approach in which we keep track of each cell, instead of continuous cell densities measured in approaches modelling the colony dynamics in terms of reaction-diffusion equations^57^. To keep the simulations sufficiently simple we also choose a non-interacting approach, such that, in principle, placed cells may overlap with already existing ones. This effect is not too pronounced, as it can be interpreted as cells growing on top of each other (the next step in volume-expanding biofilm growth)^28,58,59^.

To construct the overall concentration perceived by a single cell in the colony, we superimpose the AI fields *c*(**r**, *t*) produced by individual cells, as mediated by the reaction-diffusion equation

2 $$\frac{\partial c({\bf{r}},t)}{\partial t}=D{\nabla }^{2}c({\bf{r}},t)-\delta c({\bf{r}},t)$$

in polar coordinates, where **r** is measured from the cell centre. On top of this diffusive spreading dynamics the AI molecules degrade exponentially with rate *δ*, while we take the AI production rate *φ* to be unity in our time units (this choice merely renormalises time). As AI molecules degrade sufficiently fast, we choose natural boundary conditions for *c*(**r**, *t*) in an open system requiring the density to vanish far away, lim_*r*→∞_*c*(**r**, *t*) = 0. We refer to^29^ for the theoretical quantification of other—adsorbing and reflecting—boundary conditions on the properties of QS. For a colony in a confined area (e.g., a Petri dish), the AI concentration profile might get reflected from the boundaries and thereby affect the QS properties (especially for slowly degrading AIs and small confining domains). A multitude of approaches for the computational modelling of cell-cell communication and bacterial colony growth has been developed (see, e.g., references)^47,60–73^. They involve stochastic modelling on individual level, modelling approaches based on a variety of deterministic partial differential equations (they describe the transport properties of various molecules, mass fluxes of the components, diffusion and advection properties, etc.), or stochastic cellular-automata models, to mention but a few. Multiple aspects of QS were considered on different levels of sophistication, with a number of features of natural bacterial habitats being described.

**Figure 2:**
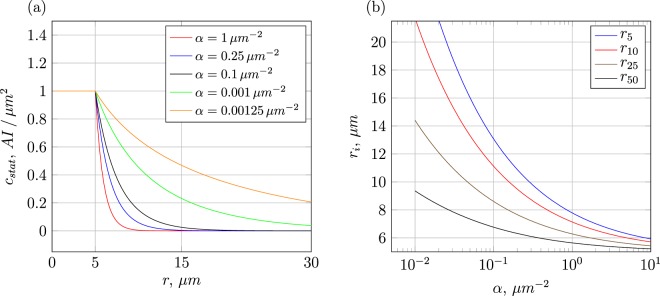
Left: Single-cell AI profile, Equation (3), for different values of the inverse signal-range parameter *α*. Right: Distances *r*_*i*_ (measured in *μ*m from the cell centre) at which the concentration has dropped to *i*% of its initial value, as function of *α*.

The solution of the AI diffusion Equation (2) rather rapidly relaxes to the steady state. We thus arrive at the time-independent single-cell AI concentration field (see Fig. 2)

3 $${c}_{{\rm{st}}}(r)=\frac{{K}_{0}(r\sqrt{\delta /D})}{{K}_{0}(2{r}_{c}\sqrt{\delta /D})}$$

in terms of the modified Bessel function *K*_0_ of the second kind (Macdonald function). Here we chose the integration constant such that the concentration is unity (in units of the induction-threshold level *β*_thr_) for the midpoint distances of two cells that just touch each other. In the case of overlapping cells, when *r* ≤ 2*r*_*c*_, the concentration is truncated to the value at $r=2{r}_{c}\sqrt{\delta /D}$. The diffusion coefficient *D* and degradation rate *δ* solely occur as the ratio

4 $$\alpha =\delta /D.$$

This parameter corresponds to the inverse square of the signal range (the typical distance reached by a diffusing molecule before degradation). Finally, the overall concentration of AIs perceived by a single cell is simply the sum of the concentration fields *c*_st_(*r*) originating from all cells of the colony. In this superposition we assume that the steady-state concentration *c*_st_(*r*) is reached much faster than the cell division time 1/*γ*.

The half-life estimates for AHL molecules, 1/*δ*, vary from 10 hours^28^ over 1 day^74^ to 7 days^29^ (in the absence of bacterial cells). However, in the case of absorbing system boundaries the depletion can be much faster: namely, of the order of the time needed for a AI molecule to diffuse across the system and get absorbed to the boundaries (some hours)^29^. These variations give rise to variations of the parameter *α*. Moreover, AI molecules get depleted by the QS-cells themselves and can be washed away by local fluid flows. The measured characteristic distances in the case of an absorbing boundary proposed in^28^ is $\sqrt{D/\delta }=3$ mm which translates to *α* = *δ*/*D* ~ 0.1/mm^2^. The values of *α* in our model vary between 10^−3^ and 10^3^/*μ*m^2^ that corresponds to the range of typical distances between ≈0.03 and ≈30 *μ*m. The separation of 30 *μ*m is indeed a typical extension of early-stage biofilms, both in QS experiments^14^ and modelling approaches^49^, while 0.03 *μ*m is clearly a very short length of spatial extensions for a bacterial colony. On the other hand, the boundaries in the current model are open and not absorbing. Finally, the measured diffusion constant of AHL molecules in agarose discs is *D* ~ 300 *μ*m^2^/sec^28,29^. In the model we set a constant, bacteria-density-independent diffusion coefficient of AI molecules (a valid approximation for the early stages of colony development). Moreover, we refer to^75^ for the absolute and relative-to-water diffusivities measured in bacterial biofilms for a number of solutes of different molecular weights. Lastly, the basal production rate of AI molecules by a single bacterial cell is in the range ~110 per sec^76^.

The initial condition is the number *n*_*c*_(*t* = 0) of seed cells, spread randomly within the fixed colony radius *R*. To reflect natural variations every sample colony starts with a different number of cells, that is, *n*_*c*_(*t* = 0) is a realisation of a Poisson-distributed random variable with

5 $$\langle {n}_{c}\rangle =\langle {n}_{c}^{2}\rangle -{\langle {n}_{c}\rangle }^{2}=\rho \pi {R}^{2}.$$

Entering the actual division loop, the mother cell for division is picked uniformly-randomly. The spawn position of the daughter cell (the other daughter cell takes up the same position as the mother cell) is determined by picking a random angle and a fixed distance *d*_new_. Alternatively, the new position of the daughter cell can be calculated using the Gaussian distribution (1), for which the degree of dispersion is modulated by *σ*_str_. Every seed cell is assigned a label that gets inherited by all daughter cells. During the colony evolution we keep track of a cell’s origin and identify “genetically related” cell families or lineages.

The next step is to calculate the AI concentration at the new cell location, to check if this cell may be induced right away. If a cell is induced its AI synthase is up-regulated by the factor (1 + *η*) leading to stronger emission of AIs^47^. After the placement of the daughter cell the AI levels of all cells are updated to check if more cells are induced. If not, the increment *t*_gap_ is added to the time counter, and a new mother cell is picked. This iteration continues until the designated termination condition is reached. Colonies with a high value of *d*_new_ or, respectively, *σ*_str_ result in somewhat larger but, importantly, more spatially homogeneous colonies due to mixing of daughter cells from different seed cells. Conversely, smaller values of these spatial homogeneity parameters lead to smaller, more densely clustered colonies with a higher degree of family separation (see below).

We study the system by variation of the inverse signal-range parameter *α*, the spatial homogeneity parameters *d*_new_ or *σ*_str_, and the surplus amount *η* of AI production upon induction. We expect that AI concentrations are higher in densely crowded areas and thus colonies with smaller spatial homogeneity have their first-induction point earlier. Conversely, it may take longer until the entire colony is induced because subcolonies can be isolated and thus cannot profit from high AI levels due to the patchiness when the signal range is limited. Otherwise, if *η* is large enough the increase in signal production of an even spatially confined induced area might, given a sufficiently large signal range, lead to a cascade inducing larger fractions of the population. To explore how far such effects indeed occur we quantify both on a single-colony level and on a statistical ensemble level.

**Figure 3:**
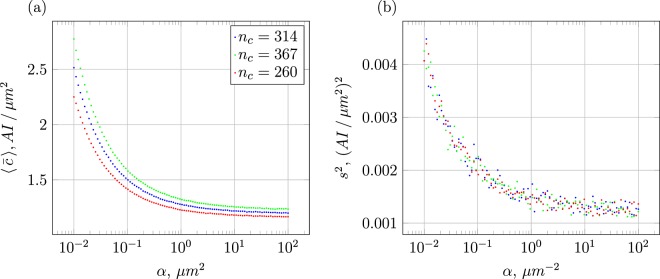
(**a**) Mean concentration $\langle \bar{c}\rangle$ as function of the inverse signal-range parameter *α* and (**b**) the corresponding sample variance *s*^2^ for an initial colony radius *R* = 200 *μ*m.

Since measuring AI levels of every cell is unrealistic experimentally, we calculate the arithmetic mean concentration $\langle \bar{c}\rangle$ of all cells as a central object for our statistical analyses. Before studying the time evolution of $\langle \bar{c}\rangle$ for a given realisation of cell colony we consider the relation of $\langle \bar{c}\rangle$ with the signal range and the number of seed cells *n*_*c*_. To this end a fixed number of randomly spread seed cells within a fixed radius *R* are chosen. The resulting $\langle \bar{c}\rangle$ as seen in Fig. 3 then shows (i) a deterministic relation to *α*. (ii) The corresponding sample variance *s*^2^ in Fig. 3 displays a similar behaviour. It describes the range of possible mean concentrations with a given signal range, that is, it helps to appraise the sensitivity of $\bar{c}$ to the exact spatial distribution of the seed cells.

While higher expected mean concentrations using configurations with smaller *α* values are immediately intuitive, the lower sample variance for higher *α* values leads to the conclusion that the concrete spatial configuration of cells is playing a minor role. Considering that large values of *α* imply an effective signal range of barely a few microns, as can be seen in Fig. 2, means that in this case variations are mostly caused by close-by or even overlapping cells. The extent of cell overlap and overall degree of spatial heterogeneity of a colony on the induction process is elaborated further when investigating the statistical data in section 4. Before doing so, we first evaluate the time evolution of single colonies, to see what parameters are relevant to classify the induction dynamics of a growing heterogeneous colony.

## Results

### Single-colony time evolution

**Figure 4:**
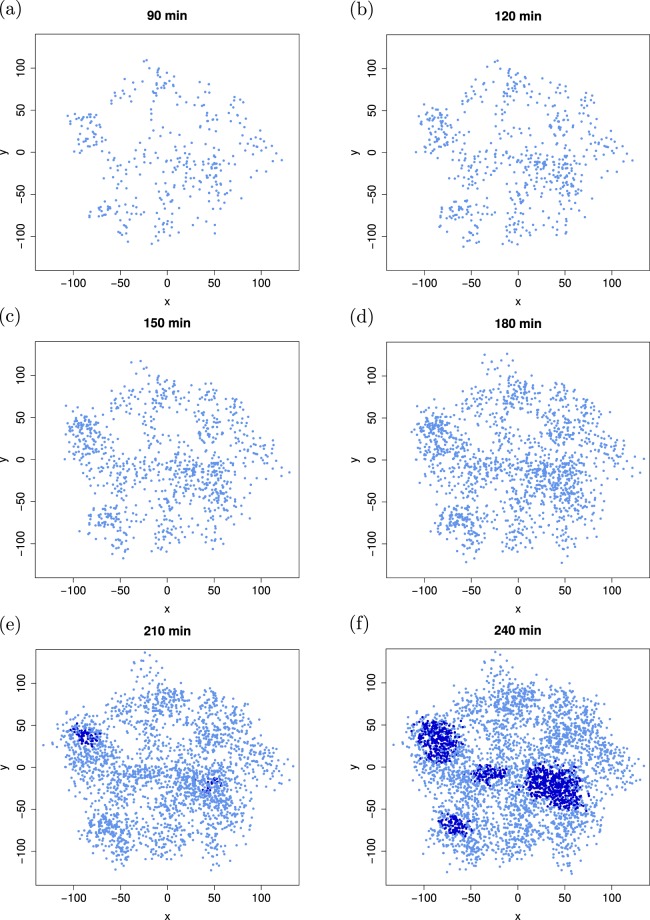
Time evolution of a growing colony with random initial cell seeding. Induced cells are rendered in dark blue while non-induced cells are shown in light blue. Parameters: *α* = 0.05/*μ*m^2^, *d*_new_ = 10 *μ*m, and *η* = 1. The first induction occurs after 192 min. The colony radii of ~100 *μ*m in our simulations agree with previous estimates/evidence^6,48,49^ (larger colonies are realised for higher *σ*_str_ values). The cell coordinates in the panels are given in *μ*m.

In Fig. 4 we show the time evolution of a growing cell colony during the time span from 90 to 240 min. We note that for different cell types or environmental conditions this time scale may need to be significantly rescaled while the effects of geometric disorder remain unaltered. In the colony of Fig. 4, due to the random placement of daughter cells denser clusters of cells emerge. At 192 min the first-induction event is recorded in the densest area, as can be seen at time 210 min (dark blue cells at the location near (−90, 40)). During the following 30 min interval several other local clusters are induced. While in this simulation the initial seed cells were placed randomly within the radius *R*, even for perfectly regularly placed seed cells an apparent randomisation emerges relatively quickly due to the random placement of daughter cells, see Fig. S.1. It is typical for most parameter choices used here that induction occurs after some 180 min, while a large part of the entire colony is induced after some 250 min.

As shown in Fig. 2 the AI concentration field of a single cell decays rapidly over a few *μ*m for the chosen values of the inverse signal-range parameter *α*. This suggests that even around very dense areas the cumulative AI concentration has a rather short range, due to degradation of AI molecules. Figure S.2 confirms this reasoning: the concentration variation depicted in the heat map for the shown bacteria colony at the moment of the first-induction event is relatively high. The concentration in areas with low cell densities is almost vanishing, in the denser regions an up to three-fold change of the concentration level can be observed. This effect allows for rather well defined, local induction regions, as opposed to an homogeneous situation as that considered in^47^.

**Figure 5:**
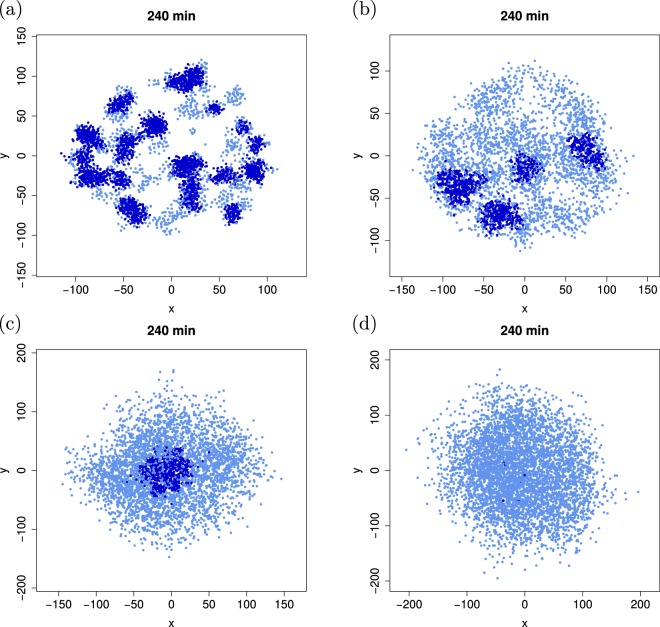
Four different colonies after 240 min time evolution with increasing spatial homogeneity *d*_new_ (*d*_new_ = 5, 10, 17.5, and 25 *μ*m for panels (a,b,c,d), respectively). In the most heterogeneous colony (panel (a)) several induction subcolonies are visible (dark colour), while in the most homogeneous case (panel (d)) only the very first-induction events are visible. Parameters: *η* = 1 and *α* = 0.25/*μ*m^2^. The coordinates in the plots are given in *μ*m.

Figure 5 demonstrates the effect of spatial disorder on the induction dynamics of the growing cell colony. When daughter cells are placed relatively close to their mother cells (*d*_new_ = 5 *μ*m) the initial inhomogeneities of the seed cluster are amplified, and significantly denser local clusters emerge. In these clusters induction occurs relatively quickly, see also the quantitative analyses below. Upon increasing the placement distance of the daughter cells up to *d*_new_ = 25 *μ*m, the colony expands significantly (note the different scales in the four panels) and, concurrently, appears much more homogeneous. In such a scenario it naturally takes longer for the first induction to occur. It will be an interesting question to address whether there eventually occurs an inversion towards full colony induction, that is, whether homogeneous colonies are completely induced earlier than heterogeneous ones.

**Figure 6:**
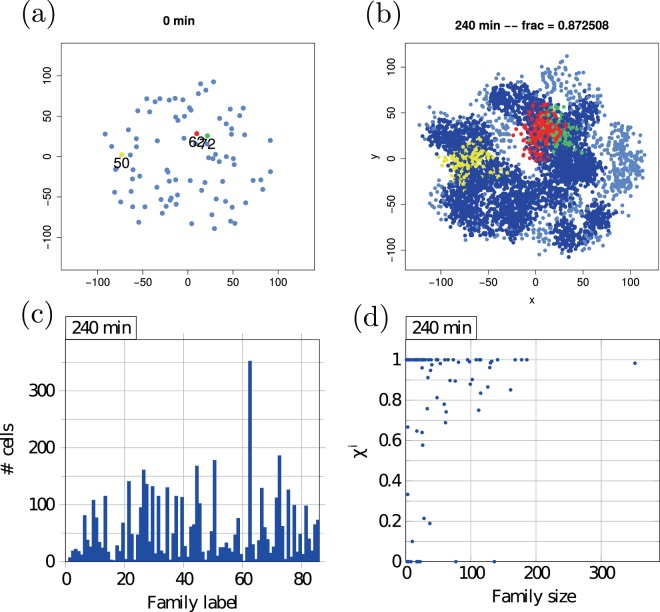
Cell-lineage data for the same colony as shown in Fig. 7. Panel (a) shows the corresponding 86 seed cells. Each seed cell gives rise to one lineage of cells, labelled *i*. The three largest families (#62, #72, and #50) after 240 min of time evolution—coloured red, green and yellow, respectively—are shown in panel (b). Coordinates in panels (a,b) are in *μ*m. Panel (c) compares the distribution of cell numbers over different cell families (the lineage is a collection of cells from the same seed). The histogram in panel (d) shows the size distribution of the 86 cell lineages. The quantity *χ*^*i*^ denotes the fraction of induced cells in family #*i* after 240 min of time evolution.

The growing colony is characterised by different quantities in Figs 6 and S.3. For the shown cell colonies the time evolution of the mean AI concentration $\bar{c}$ in Fig. S.3 demonstrates the quick increase over a few tens of minutes after the first induction at 179 min. At around 225 min the mean concentration reaches the induction level *β*_thr_. The increase of the mean concentration in this time interval exceeds the trend given by the exponential growth of cell numbers. This is due to the doubling (for the parameters chosen here) of the AI production for induced cells. The induction bursts of large numbers of cells in this dynamics are shown in Fig. S.3(c,d), where we show the fraction *χ* of induced cells along with the number *k*_*t*_ of induced cells per time step. The latter demonstrates that several induction bursts involving 20–60 cells and even a single burst of 100 cell inductions may occur.

#### Induction bursts

The most striking phenomenon of the plots in Fig. S.3 are the jumps in the mean AI concentration $\bar{c}$ and the fraction *χ* of induced cells. These jumps directly correspond to the highest peaks in the number *k*_*t*_ of cells induced at the current time step. These events represent the induction of entire clusters within a single reproduction period due to the elevated rates of transmitter production of just one or a few cells that eventually initiate the induction burst. The condition for such bursts is based on a non-negligible increase of AI production *η* upon induction of a cell. How precisely this induction behaviour is influenced by the magnitude of *η* is investigated later on.

We first visualise the burst effect in a single-colony analysis. To see the extent of induction bursts we identify the largest jumps in $\bar{c}$ and *χ* corresponding to the largest peaks of *k*_*t*_. These are compared to the average number of induced cells per time step so far. The average is taken over a sample including all division steps since the last burst event. If the difference in burst size is large enough, the algorithm colours all cells that got induced at the critical time step. For the algorithm to activate a threshold difference of 8 standard deviations to the average jump size was chosen. Figure 6 decomposes the distribution of cell family sizes and induction levels per lineage after 240 min, further detailing the burstiness of the induction process.

**Figure 7:**
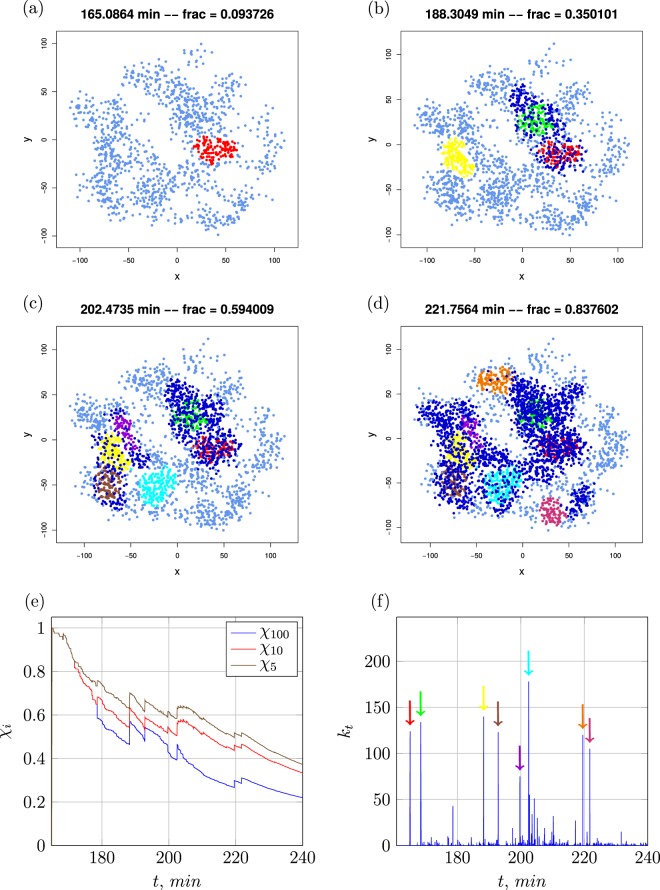
Time evolution of a single colony with coloured induction bursts, panels (a–d). All custom-coloured cells were induced within the same time step in the simulations. The quantities *χ*_*i*_ in panel (e) denote the fractions of induced cells that were induced in a burst of size *i* or larger. The number *k*_*t*_ of induced cells per time step is shown in panel (f), the coloured arrows refer to the colour coding of the colony diagrams in panels (a–d). Parameters: *d*_new_ = 7.5 *μ*m, *η* = 3.0, and *α* = 0.16/*μ*m^2^. First-induction event occurs at 170 min. The coordinates in panels (a–d) are given in *μ*m.

Examining the sample colony from Fig. 7 it is indeed possible to visualise the induction steps. The first induction in this simulation occurs after 165 min, in a densely crowded region in the lower right quadrant near the origin (0, 0), see Fig. 7(a). The first burst (124 cells induced, red) was large enough to induce almost 10% of the entire colony at this time. Only minutes later another area with 134 cells (green) was induced close to the first region. Within less than five minutes *χ* almost reached 20% of the colony. Though the induction process was quickly accelerating, it slowed down right after these two bursts since the two induced clusters are spatially quite clearly separated from the rest of the colony and thus beyond signal reach. If these two regions had been more entangled they could have likely acted as a pacemaker for the induction of other larger vicinal clusters. (Note that for a region to become induced it is not only necessary to have a high local cell density but induction probabilities are increased when other induced clusters are within signal range). After 20 more minutes the next induction period heralds. In the lower left quadrant a large region comprising 140 cells (yellow) are induced. Three major bursts follow shortly after, see Fig. 7(b,c), leading to the induction of nearly the whole quadrant. This period lasts for less than 15 min and pushes *χ* to almost 60%. This sample colony evolution also underlines that on average induction bursts appear to decline in size with time.

When looking at the colony after 240 min one can see that another large burst becomes more and more unlikely. After the induction hot spots are distributed over all regions they act as pacemaker for the induction of their vicinity surrounding. After 220 min of time evolution, see Fig. 7(d), and 50 min after the first induction a larger fraction of cells is coloured dark blue indicating that they were induced little by little, or in small bursts, see Fig. 7(e,f). This behaviour can be explained by the AI concentrations of many cells building up to the point of first induction close enough to the threshold *β*_thr_ so that just a few induced cells next to or within the cluster with additional AI secretion tip the balance towards cluster induction.

**Figure 8:**
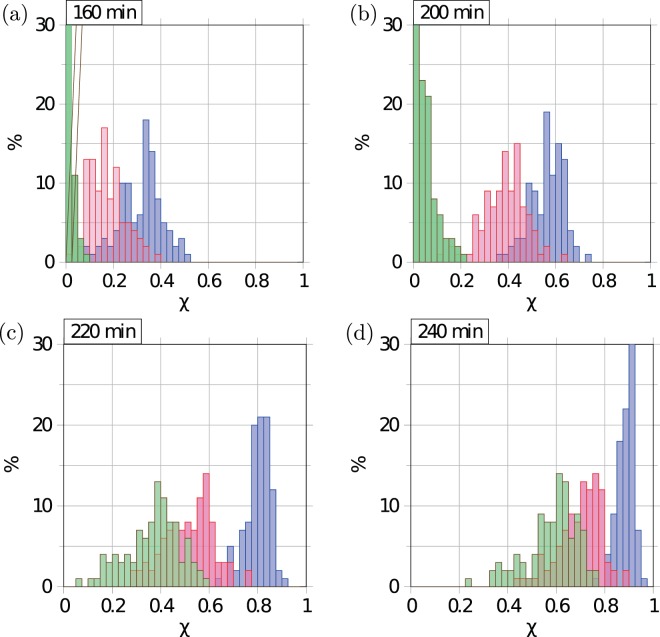
Histograms for the fraction *χ* of induced cells after different evolution times for varying standard deviation of the Gaussian distribution of daughter cell spread: *σ*_str_ = 10 *μ*m (blue), *σ*_str_ = 50 *μ*m (red), and *σ*_str_ = 100 *μ*m (green). The bars are semi-transparent to show the overlap regions. Parameters: *α* = 0.1/*μ*m^2^ and *η* = 0.5. For every data set 100 runs of the simulations were performed.

A further way to analyse the induction of the colony is depicted in Fig. 8 and S.4. At different instances of the time evolution of colonies with different degree of disorder (determined by the standard deviation *σ*_str_ of the Gaussian daughter cell displacements) the induction level is characterised by the histograms for different induction levels *χ*, as shown in Fig. 8. It can be seen that homogeneous colonies contain hardly any induced cells even at quite long times, while heterogeneous colonies already have quite advanced induction levels. However, in the course of time the homogeneous colonies quite quickly catch up, as induction occurs as a relatively sharp event, when the colony becomes critical almost everywhere. Concurrently, induction bursts increase in size with the degree of homogeneity of the colony. We show in Fig. S.4 the effect of the AI production enhancement *η* when a cell is induced. Larger values of *η* clearly speed up the induction dynamics (for the same time series of colony growth the first induction occurs irrespective of the value of *η*), while the shapes of the induction histograms are not significantly modified.

### Statistical analysis

To understand how the induction process is progressing on a statistical level we first address the conditions for the first-induction event. We quantify this by the mean first-induction time—i.e., the time that the first cell switches to the induced state—analogous to the concept of the mean first-passage time for the crossing of a prescribed value of any stochastic process. We therefore use the more common acronym MFPT. As for any given realisation the first-induction time is independent of the production increase *η* of AI molecules, the relevant parameters are the degree of spatial inhomogeneity and the inverse signal-range parameter. We will not study the effect of variations of the number *n*_*c*_ of seed cells, these are assumed to be given by the fixed parameters *ρ* = 0.0025/*μ*m^2^ and *R* = 100 *μ*m of the Poisson distribution with mean and variance (5).

**Figure 9:**
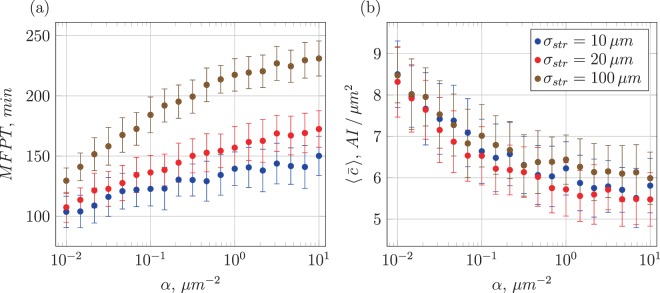
Mean first-induction time (MFPT, panel (a)) and mean AI concentration at the point of first induction (panel (b)) as function of the inverse signal-range parameter *α*. Every point corresponds to 20 runs of the simulation code.

We start with the variation of the inverse signal-range parameter *α* as shown in Fig. 9. For three different values of the standard deviation *σ*_str_ of the Gaussian daughter-cell displacement distribution, in panel (a) the mean first-induction time is seen to rise with decreasing signal range. This is quite intuitive: the further the signal penetrates the colony away from the producing cell, the more it elevates the cumulative AI concentration due to the lower degree of degradation (for the radial decay of the single-cell AI concentration profile as function of *α*, see Fig. 2). For a different spread of daughter cells, we see that for higher heterogeneity of the growing colony (smaller value of *σ*_str_) the MFPT is markedly smaller, consistent with our previous argument that in a more clustered colony denser cell regions occur, yielding higher AI concentrations.

For the mean AI concentration $\langle \bar{c}\rangle$ the decay with *α* also follows intuition: growing *α* corresponds to higher AI degradation. While for different colony heterogeneity $\langle \bar{c}\rangle$ essentially converges in the limit of small *α* (extended spreading and thus higher insensitivity to disorder) we see that the smallest degree of disorder (*σ*_str_ = 100 *μ*m) has a slightly (almost within the error bars) higher AI concentration. This is due to the significantly longer MFPT to first induction, see Fig. 9(a), such that more cells have been produced, over-compensating the dilution effect of the larger colony spread. Note that the mean concentration is a relatively bad measure to distinguish the induction threshold for different degrees of heterogeneity *σ*_str_, as shown in Fig. 9(b).

The presented data corresponds to the product of many different types of colony clusters and thus includes not only local but also cluster-cluster effects of QS, as investigated in^47^: that is, every colony here can be seen as a superposition of growing clusters of cell lineages that interact with each other in the induction dynamics. Therefore, it is useful to investigate a model colony with spatially isolated seed cells to gain further insight into the degree of locality of the induction process. For this purpose 100 cells were distributed on a grid with a distance of 200 *μ*m to each lattice neighbour. This distance is large enough so that even for very high signalling ranges (very small *α*) effectively no AI molecules arrive at another cell family. Hence, different cell families will develop independently from each other, until quite mature stages of the colony.

**Figure 10:**
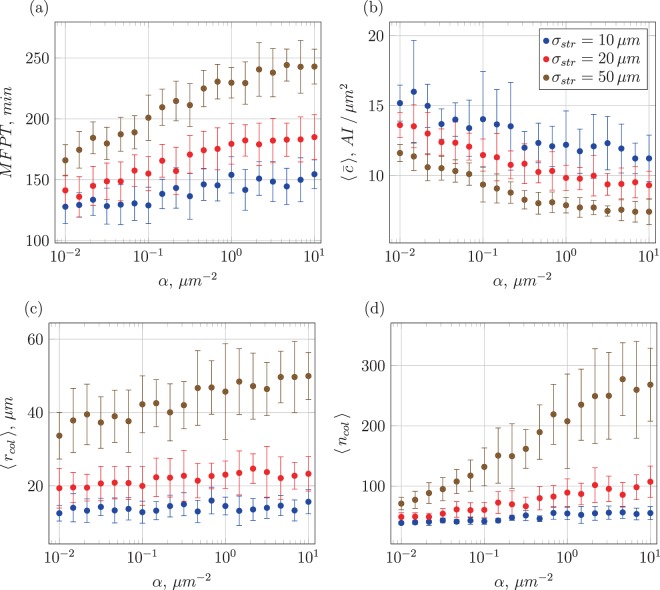
Dependence of several key quantities on *α*: mean first-induction time (**a**), mean AI concentration at time of first induction (**b**), mean cluster radius (**c**), and mean number of cells per cluster (**d**). The colour coding for different *σ*_str_ is the same as in Fig. 9. Each data point is taken from 20 simulations runs performed at *η* = 0.

In addition to the MFPT and $\langle \bar{c}\rangle$ of the first-induced cluster this simulation also delivers data on the mean span 〈*r*_col_〉, the largest distance of any cell in the cluster to the centre of mass of the cluster at the time of first induction as well as its mean number of cells 〈*n*_col_〉 (cluster size). The general trends of the colony with separated cell clusters shown in Fig. 10 are similar to those of the original system displayed in Fig. 9. On closer inspection we see that both the mean first-induction time and the mean AI concentration at the point of first induction have larger values. This fact already reflects the partial interference between clusters. The general behaviour of MFPT and $\langle \bar{c}\rangle$ in Fig. 10 is not surprising: for short signal ranges (large *α* or high degradation) the typical concentration is lower and induction on average occurs after longer times. With growing displacement *σ*_str_ the mean induction time increases, as typical distances between cells are larger. Concurrently, the mean span of the cluster grows with increasing *σ*_str_ as well as the mean cluster size. The AI concentration of the cluster containing the first induced cell is decreasing with larger *σ*_str_ as this quantity is only measured per cluster and this scheme disregards the empty space between clusters.

**Figure 11:**
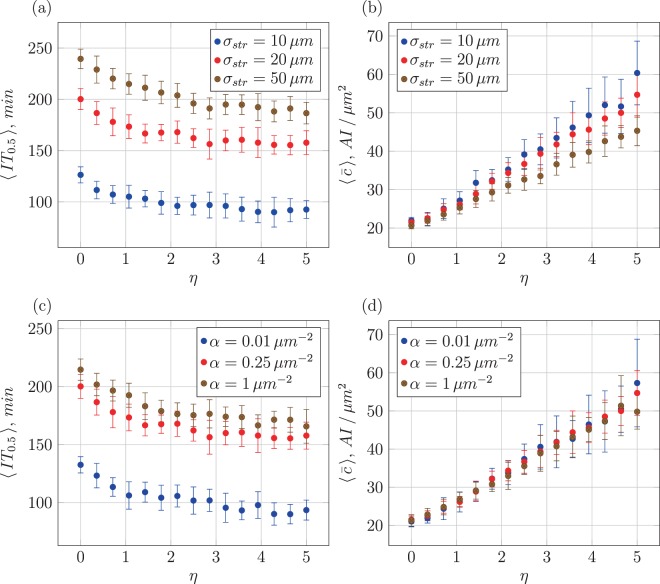
Mean times when 50% of a population have been induced and corresponding mean concentrations as function of *η* upon induction, for different inverse signal-range parameters *α* and spread of daughter cells *σ*_str_. Parameters: *β*_thr_ = 20 in units AI/*μ*m^2^, *α* = 0.25/*μ*m^2^ in panels (a,b), *σ*_str_ = 20 *μ*m in panels (c,d).

While examining family-separated clusters gives a good impression of the locality of the induction process, the properties of the mean first-induction point do not provide any insight into the later induction process, when additional AI production from already induced cells becomes relevant and the spatial arrangement of the cells starts playing an even more decisive role. To further understand what shapes the induction of new cells it is useful to define a quantity measuring the fraction of induced cells for a statistical population in addition to the fraction *χ*(*t*) of induced cells (Fig. S.3). For this purpose we consider the “order parameter” 〈IT_*χ*_〉 defined as the expected time when a fixed *χ* value (in the interval (0, 1)) is reached. For instance, 〈IT_0.5_〉 is the expected time when 50% of the entire cell population has been induced, potentially an interesting quantity to be studied in experiments. In comparison to the MFPT, 〈IT_*χ*_〉 is a function of the additional production rate *η* of AI by already-induced cells. We present results for the case *χ* = 50% in Fig. 11, further plots are gathered in the Supplement.

We first focus on the case of no surplus AI production (*η* = 0). As function of the inverse signal-range parameter the general behaviour of 〈IT_0.5_〉 in Fig. S.5 is similar to that of the MFPT in Fig. 9 but with slightly lower standard deviations. Varying the spatial homogeneity parameter *σ*_str_ similarly shows that more homogeneous colonies (larger *σ*_str_ values) take longer to reach a certain percentage of induced cells. A clearer distinction can be seen when observing the corresponding mean AI concentration in Fig. S.5(b). While in Fig. 9(b) the behaviour of $\langle \bar{c}\rangle$ shows a distinct downward trend in dependence on *α*, when higher fractions of the cells are induced the mean concentration becomes increasingly independent of *α*. This gradual disappearance of the *α* dependence can be observed when looking into the same kind of data for lower values of *χ* (Fig. S.6), as seen for *χ* = 0.25 in Fig. S.7(a). Here, the effect is weaker than for the case of the MFPT but still more significant than for the case 〈IT_0.5_〉. When larger fractions of a colony are induced and the distances between already induced cells are considerably smaller, degradation effects become less pronounced.

In more advanced colonies with higher values of *χ* another noticeable effect is taking place. While for the point of first induction (see Fig. 9(b)) the mean AI concentration hardly depends on the degree of spatial inhomogeneity set by *σ*_str_, we now see a clear separation of the $\langle \bar{c}\rangle$ values for different *σ*_str_, especially for the case *χ* = 0.75 shown in Figs S.8 and S.9. Higher *σ*_str_ leads to larger spatial extensions of the colony and thus the respective mean AI concentration is lower. Naively, more homogenous colonies would be expected to have lower mean concentrations because of their larger extension. While first induction is mostly governed by local anomalies of the cell distribution, the spatial-homogeneity parameter gains more influence on the mean concentration at later stages of colony development. Note also that we collected the data with the step of *χ* being 0.01, but present here the results only for *χ* = 0.25, 0.5, and 0.75.

Let us now address the influence of the AI production rate increase *η*. In Fig. S.5(c) as expected, a lower *η* leads to longer induction times 〈IT_*χ*_〉 while the general behaviour as function of *α* remains unaffected. The shift of the curves from *η* = 0 to *η* = 1 is larger than that from *η* = 1 to *η* = 2, pointing at a nonlinear relation between induction time and production increase *η*, as confirmed for the larger variation shown in Fig. 11. The associated mean AI concentration in Fig. S.5(d) shows clearly lower values for lower levels of additional AI production. For a lower value of *χ* in Fig. S.7(b) this trend is also observable. The standard deviations of $\langle \bar{c}\rangle$ are rising with *η* because higher additional AI production overstates the effects of denser regions on the mean AI concentration in a similar way as a longer time evolution would.

The detailed analysis of the induction time and mean concentration as function of *η* in Fig. 11 has a clear message: while the mean concentration increases almost linearly with *η*, as expected, the decrease of the induction time flattens for larger *η* values. Thus, increasing the AI amount in the positive feedback loop is mainly beneficial for induction at smaller *η* values. Too large *η* values, corresponding to costly cellular processes such as protein production, do not pay off. The influence of the spatial disorder, as shown in Fig. 11(a) in fact is more important than that of the production increase, similar to the effect of *α*. In that sense physical boundary conditions cannot be compensated by the involved biochemistry.

The associated mean AI concentrations $\langle \bar{c}\rangle$ shown in Fig. 11(b,d) increase approximately linearly with *η*. Spatially heterogeneous colonies realised at smaller values of *σ*_str_ show slightly larger values and standard deviations in Fig. 11(b). This somewhat greater variety of $\langle \bar{c}\rangle$ can be explained by the number of possible invariant spatial arrangements a system can obtain in regimes of a certain spatial homogeneity. This number is arguably larger in highly heterogeneous systems. Since denser populated regions are more likely to spawn even more new cells, every specific spatial arrangement of cells gets overstated at longer times or larger *η*. This argumentation is supported when analysing the data for lower values of *χ* in Fig. S.7(c,d) where the standard deviations are proportionally smaller; and in later time evolution in Fig. S.9(c,d) where the standard deviations are proportionally larger.

In the earlier induction process for *χ* = 0.25 in Fig. S.7(c,d) the rise of the mean AI concentration $\langle \bar{c}\rangle$ does not start right above *η* = 0. Instead, for all levels of spatial homogeneity and all signal ranges presented here it takes a greater amount of additional AI production to influence $\langle \bar{c}\rangle$. During the later time evolution of the colony with larger *χ* values, the rise of $\langle \bar{c}\rangle$ sets on already at lower values of *η*, see Fig. S.9(c,d). Colonies with higher levels of heterogeneity show slightly larger concentrations in the earlier process (though there is no clear separation given the resulting standard deviations).

The data in Fig. 11(c) where *σ*_str_ is held constant at an intermediate value and *α* is varied from curve to curve demonstrate that the induction time behaves similarly to Fig. 11(a), and it is again shifted by the variation of *α* but not influenced in its general functional form. The associated $\langle \bar{c}\rangle$ grow with *η* as well, see Fig. 11(b). We note that for *χ* = 0.25, as seen in Fig. S.7(d), colonies with large signal ranges $1/\sqrt{\alpha }$ display slightly higher levels of the mean AI concentration than colonies with small signal ranges. However, this phenomenon vanishes in higher developed colonies, see Fig. S.9(d). The effect inverts during the colony evolution: Fig. S.9(d) displays lower levels of AI concentration at small inverse signal-range parameters *α*, albeit the standard deviations for *α* = 0.01/*μ*m^2^ are the largest for all *χ* (due to a higher sensitivitsy of $\langle \bar{c}\rangle$ on the spatial arrangement of the cells in high signal-range regimes). For smaller *α* colonies have significantly shorter induction times, that is, less cells have accumulated in the colony when a certain fraction got induced.

The same argument can be used for the variation of *σ*_str_. High signal range colonies are more likely to develop a greater number of independent hot spots earlier in the induction process and therefore reach higher mean concentrations earlier. This advantage, however, vanishes over time when lower signal range colonies catch up due to sheer numbers of cells and a higher degree of overlap. This is one of the central results of the current analysis.

The final remaining analysis is the detailed variation of *σ*_str_ with varying values of *α* and *η*. Holding the signal-range constant and using different values of *η* leads to the results displayed in Fig. S.10. Naturally higher *η* lead to earlier induction of many cells. The nonlinear effect of increasing *η* is confirmed in this data series. The time 〈IT_*χ*_〉 grows with the spatial homogeneity parameter in Fig. S.10(a). This behaviour can be seen as well for *χ* = 0.25 in Fig. S.6(e) at lower, and for *χ* = 0.75 in Fig. S.8(e) for higher induction levels of the entire colony. The corresponding mean AI concentrations exhibit lower magnitudes for lower values of *η*, as expected. The mean concentrations decrease with *σ*_str_ and depend on the additional AI production, see Fig. S.10(b). Higher levels of *η* show the strongest relative decline of the mean AI concentration with *σ*_str_. The standard deviations grow with time, similar to the data for *χ* = 0.25 and *χ* = 0.75. The growth of standard deviations for low values of *σ*_str_ present for all degrees of induction may be explained by the fact that highly heterogeneous colonies usually consist of well-separated clusters and therefore have more possible spatial arrangements of clusters leading to greater variety.

Additionally a slight increase of $\langle \bar{c}\rangle$ can be observed at intermediate levels of spatial homogeneity and higher amounts of additional AI production in the earlier induction process. On varying *α* the sensitivity of the induction time on the signal range is again present, the general trend being merely shifted by the variation of *α*. Comparing with the trends for *χ* = 0.25 in Fig. S.7(f) and *χ* = 0.75 in Fig. S.9(f), at earlier induction stages no clear dependence on *σ*_str_ can be identified while at higher signal ranges higher mean concentrations abound. At higher induction levels the mean concentration of high-signal ranges steadily declines relative to the lower signal-range regime, until at the late stages colonies with a low *α* exhibit the lowest concentrations, similar to the description before. In the later time evolution a decline of the mean concentration as function of *σ*_str_ is visible, see Fig. S.9(f). The effect is more distinct in low signal-range regimes though the standard deviations are significant in all cases.

#### Phase transition

**Figure 12:**
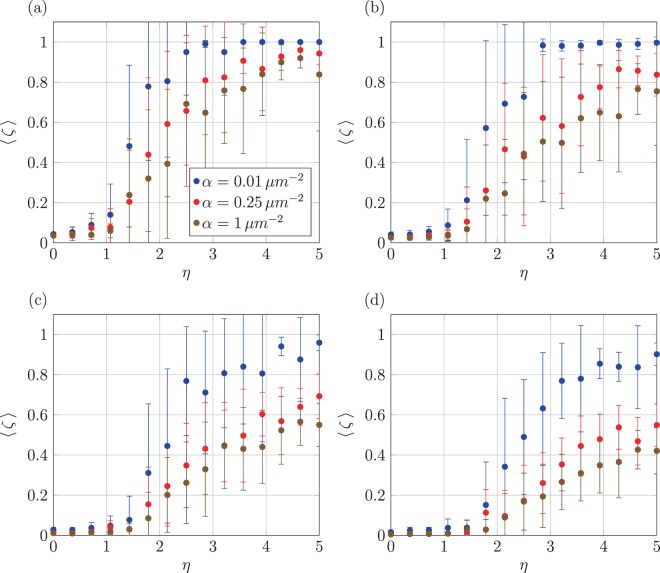
Mean relative burst size 〈*ζ*〉 as function of additional AI rate *η* on cell induction, for different levels of the daughter cell spread *σ*_str_ and for different inverse signal-range parameters *α*. Fifteen simulations runs were used for every data point. Parameters: *φ* = 1 (in units of 1/sec), *R* = 100 *μ*m, *ρ* = 0.0025/*μ*m^2^, *γ* = 1/[60 min], and *σ*_str_ = 10, 20, 50, and 100 *μ*m in panels (a,b,c,d), respectively.

Since the production of AIs is positively up-regulated after a threshold amount is reached, the vicinity of an induced cell is more likely to get induced, as well, due to elevated concentration of signalling molecules. As seen in the above discussion burst induction is typically a local phenomenon. To emphasise this point we finally study the process of burst induction for a system with a single seed cell. The central order parameter to classify the burst behaviour is the mean relative size 〈*ζ*〉 of a burst divided by the entire number of cells in the emerging colony at the time of first induction. The main effect we study here is the influence of the up-regulation quantified by *η*. The resulting data are shown in Fig. 12. We also calculated the expected number of cells in the burst, see Fig. S.11.

Figure 12 shows that while for *η* = 0 hardly more than a few cells are comprised in a burst, with increasing *η* it becomes more and more likely that an induction burst will comprise significantly larger fractions of the cluster, until the single burst becomes almost global, inducing a large fraction of cells in the cluster. This quite sharp behaviour is reminiscent of a phase transition (or percolation): it is most pronounced in Fig. 12(a,b) for smaller spreads of the daughter cell displacements as well as for the smallest inverse signal-range parameter (longest signal range), in agreement with intuition. The data of Fig. 12 also show that spatially heterogeneous colonies start their phase transition at lower values of *η* and that their upper limit 〈*ζ*〉 is closer to 1 (or even exactly one when the signal range is sufficiently high). A larger signal range—corresponding to smaller values of *α*—always leads to a higher upper limit for all levels of spatial homogeneity.

## Discussion

QS enables simple organisms such as bacteria to sample information on the density of same-species or different-species organisms in their local environment. QS controls collective actions of bacterial colonies via production, sensing of, and response to AI molecules. This establishes cell-cell communication and orchestrates collective/communal behaviors of bacterial colonies (sociobiology)^10,13^. QS cascades and reactions at heterogeneous conditions of bacterial biofilms^18^ emerge in terms of bacterial density, distribution of nutrients^9^, as well as AI diffusion characteristics. The dynamics of QS under constraints in natural biofilms as well as with fluid flows is important to consider on a model level. Constant flows can wash AIs away thus repressing QS. These and other “external” factors enable genetically identical bacteria to respond to signals and operate individually in different space locations in the colony and at different times. Certain cavities, and crevices of the bacteria-growth medium also impact QS^18^.

We studied the influence of the degree of spatial disorder on the induction dynamics of a growing bacteria colony, as a prototype model for early biofilm formation based on QS when the bacteria mainly form a monolayer on a surface. To this end we set up a Monte Carlo simulations scheme and studied several core quantities such as the shapes of the emerging colonies, their bursty induction behaviour, and the mean AI concentration at the point of induction. We investigated these effects as function of several core parameters: the inverse signal-range parameter measuring the decay of a single cell AI concentration field, the production increase *η* of AIs upon induction, and the distance by which a daughter cell is displaced from the site of the mother cell. We find that spatial disorder is a main factor in colony growth and induction dynamics. Our results, therefore, add to the current discussion of heterogeneity effects in QS colonies^77,78^.

Our main findings are the highly local induction of clusters in scenarios when an initial colony with random cell placement grows under conditions of short signal range—that is, when the AI concentration field emitted by a given cell falls off over the range of few *μ*m—and relatively local placement of the daughter cells. In this scenario the concentration field becomes highly heterogeneous and the local AI concentration in a given cluster grows much quicker than in the corresponding homogeneous scenario: consequently, the first induction occurs considerably earlier, a new effect observed in this study. We showed that for the studied model parameters this scenario is robust even when the seed cells are evenly spaced: the mixing due to daughter cell placement leads to a disordered geometry of the colony.

A characteristic feature of the induction dynamics is the occurrence of bursts, the simultaneous induction of a number of cells in a neighbourhood. The statistic of bursts turns out to depend crucially on the degree of spatial disorder and the signal range given by the AI degradation. Studied as function of the additional AI production rate, the burst size may show a clear, relatively sharp phase transition from very small bursts to bursts with sizes of the order of the colony size.

Our analysis introduced a family tree such that each cell carries the label of the specific seed cell it stems from. This way simulations can be used to study the degree of overlap between different lineages and effects of the exact growth and induction on a family level. Similarly, we introduced a colour labelling of clusters induced by successive induction cascades. In experiments, it should be possible to endow different cell lines with different colours or other labels. While this may not be possible for large seed colonies in the hundreds, it should be possible to distinguish, say, of the order of ten families in the course of the biofilm formation.

The progress of the colony induction was monitored using different quantities. In addition to the AI concentration and the first-induced cell cluster we introduced the fraction of induced cells at a given snapshot of the colony, as well as the mean first-induction time and the typical times when a given fraction of the colony is induced. Moreover, we studied the distribution of induction levels as function of time, showing distinct features depending on the physical parameters of the system. In particular, for given parameters it may happen that while highly heterogeneous colonies are induced much earlier than homogeneous ones, to induce the entire colony it may take longer for the heterogeneous case as major voids in cell distribution have to be overcome upon AI-signal propagation.

An interesting question for biological systems is the economy of resources. Thus, gene expression and molecular signalling processes in cells are typically performed by passive diffusion^79^, as active transport would easily exceed the resources of a cell. The often extremely low concentrations of signalling molecules may be compensated by spatial proximity (geometry-control)^80^ between the loci where the molecules are produced and where they are supposed to bind^81–84^. For the QS scenario we here showed that excessive production of AIs may not lead to more reliable signalling and, thus, allows a similar housekeeping economy for the partaking cells. While the positive feedback loop leading to an up-regulation in the AI production in an induced cell clearly increased the induction dynamics of a growing colony, for even higher up-regulation levels the gain decreases significantly. How much this statement varies with different scenarios of spatial disorder remains to be seen.

The effects of spatial disorder studied here are relatively moderate, taking typical daughter cell displacements of few tens of *μ*m into account, but may provide decisive advantages for cell types with more optimised QS strategies. For mobile daughter cells two scenarios might be considered: (i) cells may actively seek for either already dense or empty areas, thus emphasising or moderating the effects studies here. (ii) Or, cells may choose different, random placement such as long-tailed distributions as used in the location of sparse resources by many animals as well as bacteria^85,86^. Note that even when cells move in a highly randomised fashion, effects such as bulk mediation may lead to non-Gaussian surface displacements at intermediate scales^87^. It should be interesting to see how such scenarios balance the induction versus the spreading behaviour of colony formation by QS. Ultimately, such questions are central for the study of locality versus globality effects in molecular signalling between bacterial cells^88^ and in similar systems.

Additional important aspects to study include different, potentially anisotropic dispersal, proliferation, and motility strategies of bacterial cells^8^, non-Fickian or anomalous diffusion of AI molecules^84^, as well as the diffusivity of AI molecules depending on local bacteria density (transport channels and porosity of a biofilm)^8,61,63^. Other biologically-relevant aspects should include stimulations of cell mortality (when local cell densities become too high to allow flows of nutrients and oxygen), disposal of poisonous metabolic/waste products, of the history of experiencing cell environments^36^, mimicking heterogeneous micro-scale structure of bacterial biofilms/colonies^9,10^, and QS mechanisms for multiple species of QS-signalling molecules (“interference”)^14^. Modelling environment-induced changes in the gene-expression levels and formation of bacterial subpopulations (“phenotypical heterogeneity”)^9^ are also important to consider.

From the modelling perspective, the effects of excluded volume in dense bacterial colonies will affect diffusion of small molecules through voids and spaces between cells. Naturally, the growth preferences of a bacterial colony from a single seed (provided by a daughter cell) are expected to depend on cell density around the point of first-cell placement as well as on availability of nutrients there. The behaviour of a more complicated model under the conditions of limited amount of nutrients, crowding-dependent growth dynamics and division rates of bacterial cells, and with crowding-dependent diffusion of QS-inducing AI molecules will be interesting to examine. For instance, a constant diffusivity of AI molecules was used in the current analysis, but in denser assemblies of bacteria the cell density will have an effect on *D*. Our studies of tracer diffusion in crowded media with non-inert obstacles^89^ and non-homogeneous crowding^90^ have already demonstrated that the effects on free-space tracer diffusivity can be rather dramatic.

Moreover, competition for space, oxygen (aerobic and anaerobic metabolism), and nutrients^10,14,49,70,91^ between different cell species with different traits (such as size, spreading properties, or cell-division rates) will give rise to a rich behaviour, for instance, “rock-paper-scissor” competition scenarios^39–41^, including inter-species “cross-talk”^50^. We refer to^9–11,14^ for the discussion of certain evolutionary aspects of QS (relative fitness benefits), selection strategies, and competition-versus-cooperation strategies for public goods in multispecies bacterial colonies. We also refer to^92^ for the mechanisms of cooperation, adaptation, and conflict in microbial biofilms.

Naturally, a certain degree of stochasticity of the model parameters is going to quantitatively affect the outcomes and predictions of the current modelling. Importantly, for the cell-division strategies different from the one with a constant rate of division (“timer”)— such as the “adder” or “sizer” scenarios^93–95^—the spreading dynamics of a bacterial colony as well as the features of the induction process and general collective behaviour of cells are expected to be affected as well.

While the modelling approach based on individual cells pursued here is very flexible and can accommodate many different features, approaches based on differential equations for chemical concentrations have the advantage that, at least in some limits, analytical solutions can be obtained^26,47^. In addition to these two strategies network-based approaches have been suggested^96^. We finally note that some of the results observed here may have relevance in morphogen-gradient based problems, in which the spatial distribution of the concentration of stimulating molecules can trigger a certain response of tissues.

## Supplementary information

Supplementary Information

## Data Availability

The data sets generated during and/or analysed during the current study are available from the corresponding author on reasonable request.

## References

[1] Costerton JW, Lewandowski Z, Caldwell DE, Korber DR, Lappin-Scott HM (1995). Microbial biofilms. Annu. Rev. Microbial..

[2] Davies DG (1998). The involvement of cell-to-cell signals in the development of a bacterial biofilm. Science.

[3] Costerton JW, Stewart PS, Greenberg EP (1999). Bacterial biofilms: a common cause of persistent infections. Science.

[4] O’Toole G, Kaplan HB, Kolter R (2000). Biofilm formation as microbial development. Annu. Rev. Microbiol..

[5] Stewart PS, Costerton JW (2001). Antibiotic resistance of bacteria in biofilms. Lancet.

[6] Donlan RM, Costerton JW (2002). Biofilms: survival mechanisms of clinically relevant microorganisms. Clin. Microbiol. Rev..

[7] Davies D (2003). Understanding biofilm resistance to antibacterial agents. Nature Rev. Drug Discov..

[8] Hall-Stoodley L, Costerton JW, Stoodley P (2004). Bacterial biofilms: from the natural environment to infectious diseases. Nature Rev. Microbiol..

[9] Stewart PS, Franklin MJ (2008). Physiological heterogeneity in biofilms. Nature Rev. Microbiol..

[10] Nadell CD, Xavier JB, Foster KR (2009). The sociobiology of biofilms. FEMS Microbiol. Rev..

[11] Nadell CD, Drescher K, Foster KR (2016). Spatial structure, cooperation and competition in biofilms. Nature Rev. Microbiol..

[12] Bassler B (1999). How bacteria talk to each other: regulation of gene expression by quorum sensing. Curr. Opin. Micorbiol..

[13] Bassler BL, Losick R (2006). Bacterially speaking. Cell.

[14] Hense BA (2007). Does efficiency sensing unify diffusion and quorum sensing?. Nature Rev. Microbiol..

[15] Waters CM, Bassler BL (2005). Quorum sensing: cell-to-cell communication in bacteria. Ann. Rev. Cell Dev. Biol..

[16] Parsek MR, Greenberg EP (2005). Sociomicrobiology: the connections between quorum sensing and biofilms. Trends in Microbiol..

[17] Atkinson S, Williams P (2009). Quorum sensing and social networking in the microbial world. J. R. Soc. Interface.

[18] Mukherjee S, Bassler BL (2019). Bacterial quorum sensing in complex and dynamically changing environments. Nature Rev. Microbiol..

[19] Nealson K, Platt T, Hastings JW (1970). Cellular control of the synthesis and activity of the bacterial luminescent system. J. Bacteriol..

[20] Miller MB, Bassler BL (2001). Quorum sensing in bacteria. Ann. Rev. Microbioly..

[21] Ahmer BM (2004). Cell-to-cell signalling in *Escherichia coli* and *Salmonella enterica*. Mol. Microbiol..

[22] Keller L, Surette MG (2006). Communication in bacteria: an ecological and evolutionary perspective. Nature Rev. Microbiol..

[23] Popat, R., Cornforth, D. M., McNally, L. & Brown, S. P. Collective sensing and collective responses in quorum-sensing bacteria. *J. R. Soc. Interface* **12**, 20140882 (2015). 10.1098/rsif.2014.0882 PMC4305403 25505130

[24] Hense BA, Schuster M (2015). Core principles of bacterial autoinducer systems. Microbiol. Molec. Biol. Rev..

[25] Whitely M, Diggle SP, Greenberg EP (2017). Progress in and promise of bacterial quorum sensing research. Nature.

[26] Müller J, Kuttler C, Hense BA (2008). Sensitivity of the quorum sensing system is achieved by low pass filtering. Biosystems.

[27] Fancher S, Mugler A (2017). Fundamental limits to collective concentration sensing in cell populations. Phys. Rev. Lett..

[28] Marenda M, Zanardo M, Trovato A, Seno F, Squartini A (2016). Modeling quorum sensing trade-offs between bacterial cell density and system extension from open boundaries. Sci. Rep..

[29] Trovato A (2014). Quorum vs. diffusion sensing: a quantitative analysis of the relevance of absorbing or reflecting boundaries. FEMS Microbiol. Lett..

[30] Li Y-H, Tian X (2012). Quorum sensing and bacterial social interactions in biofilms. Sensors.

[31] Diggle SP, Crusz SA, Cámara M (2007). Quorum sensing. Curr. Biol..

[32] Cezairliyan B, Ausubel F (2017). Investment in secreted enzymes during nutrient-limited growth is utility dependent. Proc. Natl. Acad. Sci. USA.

[33] Engebrecht J, Nealson K, Silverman M (1983). Bacterial bioluminescence: isolation and genetic analysis of functions from *Vibrio fischeri*. Cell.

[34] Smith RS, Iglewski BH (2003). *P. aeruginosa* quorum-sensing systems and virulence. Curr. Opin. Microbiol..

[35] Erickson DL (2002). *Pseudomonas aeruginosa* quorum-sensing systems may control virulence factor expression in the lungs of patients with cystic fibrosis. Infection & Immunity.

[36] Moreno-Gámez S (2017). Quorum sensing integrates environmental cues, cell density and cell history to control bacterial competence. Nature Comm..

[37] Shih P-C, Huang C-T (2002). Effect of quorum sensing deficiency on *Pseudomonas aeruginosa* biofilm formation and antibiotic resistance. J. Antimicr. Chemother..

[38] Polonsky M (2018). Induction of CD4 T cell memory by local cellular collectivity. Science.

[39] Hibbing ME, Fuqua C, Parsek MR, Peterson SB (2010). Bacterial competition: surviving and thriving in the microbial jungle. Nature Rev. Microbiol..

[40] Kerr B, Riley MA, Feldman MW, Bohannan BJM (2002). Local dispersal promotes biodiversity in a real-life game of rock-paper-scissors. Nature.

[41] Reichenbach T, Mobilia M, Frey E (2007). Mobility promotes and jeopardizes biodiversity in rock-paper-scissors games. Nature.

[42] Jamal M (2018). Bacterial biofilm and associated infections. J. Chin. Med. Assoc..

[43] Høiby N, Bjarnsholt T, Givskov M, Molinc S, Ciofu O (2010). Antibiotic resistance of bacterial biofilms. Int. J. Antimicr. Agents.

[44] Hong SH (2012). Synthetic quorum-sensing circuit to control consortial biofilm formation and dispersal in a microfluidic device. Nature Comm..

[45] Reuter K, Steinbach A, Helma V (2016). Interfering with bacterial quorum sensing. Perspect. Med. Chem..

[46] Geske GD, Wezeman RJ, Siegel AP, Blackwell HE (2005). Small molecule inhibitors of bacterial quorum sensing and biofilm formation. J. Am. Chem. Soc..

[47] Yusufali TI, Boedicker JQ (2016). Spatial dispersal in bacterial colonies induces a phase transition from local to global quorum sensing. Phys. Rev E.

[48] Melke P, Sahlin P, Levchenko A, Jönsson H (2010). A cell-based model for quorum sensing in heterogeneous bacterial colonies. PLoS Comput. Biol..

[49] Hense BA, Müller J, Kuttler C, Hartmann A (2012). Spatial heterogeneity of autoinducer regulation systems. Sensors (Basel).

[50] Silva KPT, Chellamuthu P, Boedicker JQ (2017). Quantifying the strength of quorum sensing crosstalk within microbial communities. PLoS Comp. Biol..

[51] Cornforth DM (2014). Combinatorial quorum sensing allows bacteria to resolve their social and physical environment. Proc. Natl. Acad. Sci. USA.

[52] Case RJ, Labbate M, Kjelleberg S (2008). AHL-driven quorum-sensing circuits: their frequency and function among the Proteobacteria. ISME J..

[53] Dong YH (2001). Quenching quorum-sensing-dependent bacterial infection by an N-acyl homoserine lactonase. Nature.

[54] Rajput A, Kaur K, Kumar M (2015). SigMol: repertoire of quorum sensing signaling molecules in prokaryotes. Nucl. Acids Res..

[55] Guttenplan SB, Kearns DB (2013). Regulation of flagellar motility during biofilm formation. FEMS Microbiol. Rev..

[56] Plank MJ, Simpson MJ (2012). Models of collective cell behaviour with crowding effects: comparing lattice-based and lattice-free approaches. J. Roy. Soc. Interface.

[57] Kuttler, C. Reaction-Diffusion Equations and Their Application on Bacterial Communication, In *Handbook of Statistics* **37**, 55, (Elsevier, Amsterdam) (2017).

[58] Hartmann R (2019). Emergence of three-dimensional order and structure in growing biofilms. Nature Phys..

[59] Beroz F (2018). Verticalisation of bacterial biofilms. Nature Phys..

[60] Wanner. O, Gujer W (1986). A multispecies biofilm model. Biotechnol. Bioeng..

[61] Wanner O, Reichert P (1996). Mathematical modeling of mixed-cuIture biofilms. Biotechn. Bioenging..

[62] Wanner, O. *et al*. Mathematical modeling of biofilms. *IWA Publishing, London* (2006).

[63] van Loosdrecht MCM, Heijnen J, Eberl H, Kreft J, Picioreanu C (2002). Mathematical modelling of biofilm structures. Antonie van Leeuwenhoek.

[64] Kreft JU, Booth G, Wimpenny JWT (1998). BacSim, a simulator for individual-based modelling of bacterial colony growth. J. Microbiol..

[65] Kreft JU, Picioreanu C, Wimpenny JWT, van Loosdrecht MCM (2001). Individual-based modeling of biofilms. J. Microbiol..

[66] Picioreanu C, van Loosdrecht MCM, Heijnen JJ (2001). Mathematical modeling of biofilm structure with a hybrid differential-discrete cellular automaton approach. J. Biotechn. Bioeng..

[67] Dockery JD, Keener JP (2001). A mathematical model for quorum sensing in *Pseudomonas aeruginosa*. Bull. Math. Biol..

[68] Eberl HJ, Parker DF, van Loosdrecht MCM (2001). A new deterministic spatio-temporal continuum model for biofilm development. J. Theor. Med..

[69] Chopp, D. L. Simulating bacterial biofilms. In “Deformable Models”. Edited by: Suri, J. S., Farag, A. A.; “*Biomedical and Clinical Applications*”, pp. 1–31, Springer (2007).

[70] Rahman KA, Sudarsan R, Eberl HJ (2015). A mixed-culture biofilm model with cross-diffusion. Bull. Math. Biol..

[71] Goryachev AB (2011). Understanding bacterial cell-cell communication with computational modeling. Chem. Rev..

[72] Ward JP (2001). Mathematical modelling of quorum sensing in bacteria. Math. Medicine & Biol. IMA.

[73] Klapper I, Dockery J (2010). Mathematical description of microbial biofilms. SIAM Rev..

[74] Schaefer AL, Hanzelka BL, Parsek MR, Greenberg EP (2000). Detection, purification, and structural elucidation of the acylhomoserine lactone inducer of *Vibrio fischeri* luminescence and other related molecules. Methods Enzymol..

[75] Stewart PS (2003). Diffusion in biofilms. J. Bacteriol..

[76] Alberghini S (2009). Consequences of relative cellular positioning on quorum sensing and bacterial cell-to-cell communication. FEMS Microbiol. Lett..

[77] Bauer M, Knebel J, Lechner M, Pickl P, Frey E (2017). Ecological feedback in quorum-sensing microbial populations can induce heterogeneous production of autoinducers. eLife.

[78] Gao M (2016). A crucial role for spatial distribution in bacterial quorum sensing. Sci. Rep..

[79] von Hippel PH, Berg OG (1989). Facilitated target location in biological systems. J. Biol. Chem..

[80] Godec A, Metzler R (2016). Universal proximity effect in target search kinetics in the few encounter limit. Phys. Rev. X.

[81] Kolesov G, Wunderlich Z, Laikova ON, Gelfand MS, Mirny LA (2007). How gene order is influenced by the biophysics of transcription regulation. Proc. Natl. Acad. Sci. USA.

[82] Pulkkinen O, Metzler R (2013). Distance matters: the impact of gene proximity in bacterial gene regulation. Phys. Rev. Lett..

[83] Grebenkov D, Metzler R, Oshanin G (2018). Strong defocusing of molecular reaction times: geometry and reaction control. Comm. Chem..

[84] Kar P, Cherstvy AG, Metzler R (2018). Acceleration of bursty multi-protein target-search kinetics on DNA by colocalisation. Phys. Chem. Chem. Phys..

[85] Palyulin VV, Chechkin AV, Metzler R (2014). Lévy flights do not always optimize random blind search for sparse targets. Proc. Natl. Acad. Sci. USA.

[86] Ariel G (2015). Swarming bacteria migrate by Lévy walk. Nature Comm..

[87] Chechkin AV, Zaid IM, Lomholt MA, Sokolov IM, Metzler R (2012). Bulk-mediated diffusion on a planar surface: full solution. Phys. Rev. E.

[88] Venturi V, Kerényi A, Reiz B, Bihary D, Pongor S (2010). Locality versus globality in bacterial signalling: can local communication stabilize bacterial communication?. Biol. Direct.

[89] Ghosh SK, Cherstvy AG, Metzler R (2015). Non-universal tracer diffusion in crowded media of non-inert obstacles. Phys. Chem. Chem. Phys..

[90] Ghosh SK, Cherstvy AG, Grebenkov DS, Metzler R (2016). Anomalous, non-Gaussian tracer diffusion in crowded two-dimensional environments. New J. Phys..

[91] Paul Rajorshi, Ghosh Tanushree, Tang Tian, Kumar Aloke (2019). Rivalry in Bacillus subtilis colonies: enemy or family?. Soft Matter.

[92] Xavier JB, Foster KR (2007). Cooperation and conflict in microbial biofilms. Proc. Natl. Acad. Sci. USA.

[93] Willis L, Huang KC (2017). Sizing up the bacterial cell cycle. Nature Rev. Microbiol..

[94] Westfall CS, Levin PA (2017). Bacterial cell size: multifactorial and multifaceted. Annu. Rev. Microbiol..

[95] Ghusinga KR, Vargas-Garcia CA, Singh A (2016). A mechanistic stochastic framework for regulating bacterial cell division. Sci. Rep..

[96] Yusufaly TI, Boedicker JQ (2017). Mapping quorum sensing onto neural networks to understand collective decision making in heterogeneous microbial communities. Phys. Biol..

