# Supplementary material for: Burst statistics in an early biofilm quorum sensing model: the role of spatial colony-growth heterogeneity
Source: Sci Rep. 2019 Aug 19;9:12077. doi: 10.1038/s41598-019-48525-2 (PMC6700081; doi:10.1038/s41598-019-48525-2)
Supplement: Supplementary file 1 — Supplementary Information [file 41598_2019_48525_MOESM1_ESM.pdf]

Supplementary material
Burst statistics in an early biofilm quorum sensing model: the role of spatial colony-growth heterogeneity

Oliver Kindler,[1] Otto Pulkkinen,[2] Andrey G. Cherstvy,[1] and Ralf Metzler[1]

[1] *Institute for Physics & Astronomy, University of Potsdam, D-14476 Potsdam-Golm, Germany*
[2] *Institute for Molecular Medicine Finland and Helsinki Institute for Information Technology, University of Helsinki, FI-00014 Helsinki, Finland*

We here collect the Supplementary figures referred to in the main text.

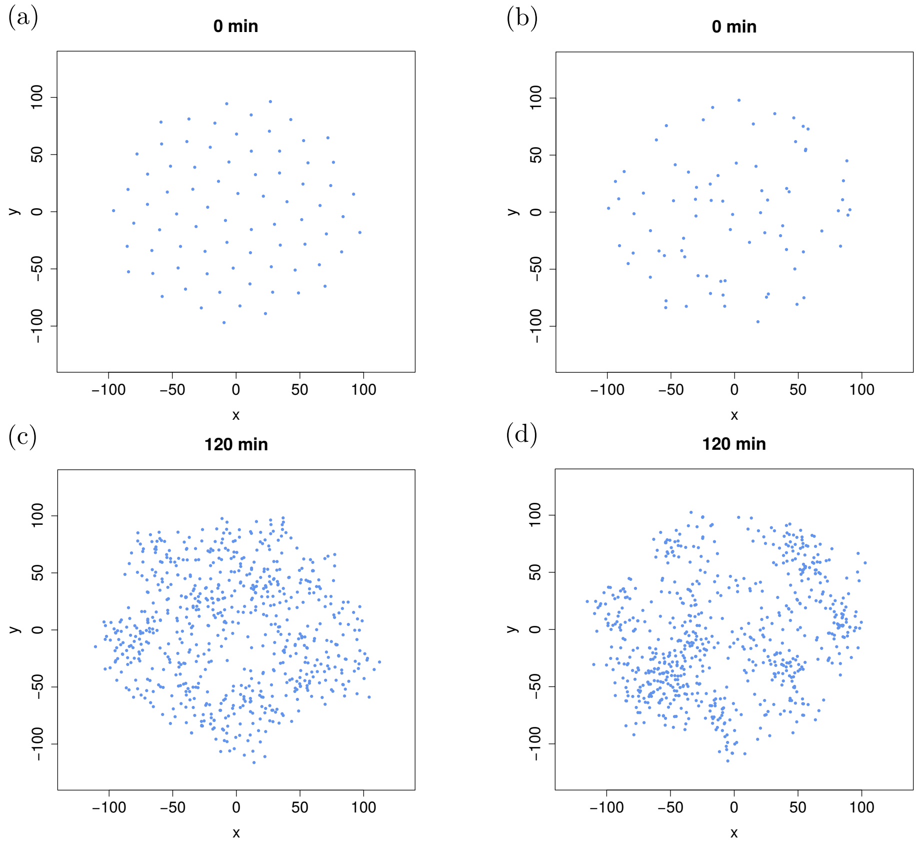

Figure S.1: Time evolution of a colony with regular (left) and random (right) initial seeding: after time evolution of 120 min both cases become almost equally randomised due to random placement of daughter cells. This trend increases with time. Parameters: $\alpha = 0.05/\mu\text{m}^2$ and $d_{\text{new}} = 10\ \mu\text{m}$. The coordinates in plots are given in μm.

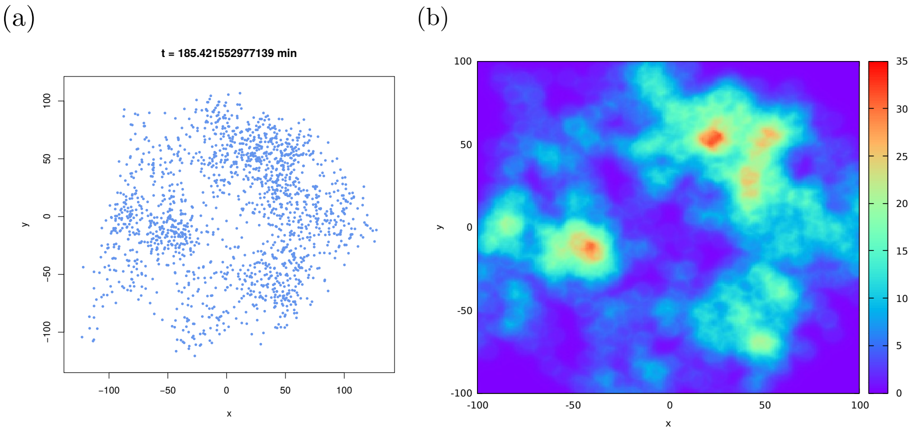

Figure S.2: Heat map (panel (a)) showing the AI concentration at the moment of first induction for the colony configuration shown in panel (b). Parameters: $\alpha = 0.25/\mu\text{m}^2$, $d_{\text{new}} = 10\ \mu$m, and $\eta = 1$. The coordinates in plots are in μm.

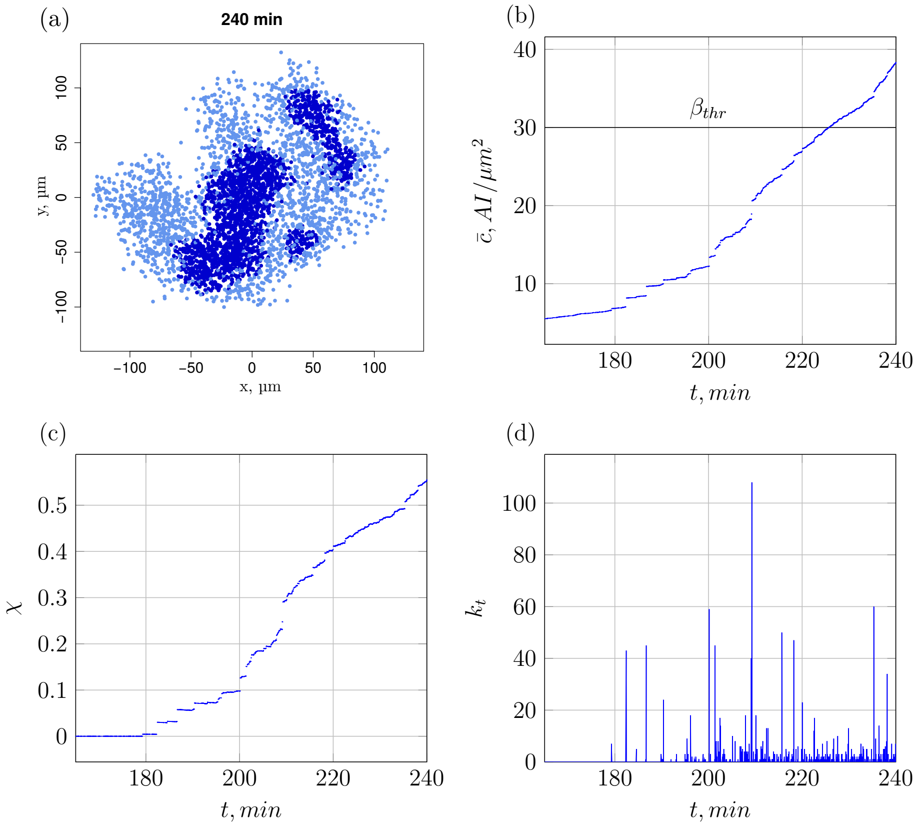

Figure S.3: Panel (a): Sample colony after 240 min of time evolution in which non-induced cells are coloured in light blue, while induced cells appear in dark blue. Panel (b): Mean AI concentration $\bar{c}$ of the single colony as function of time. Panels (c) and (d) depict fraction χ of induced cells and number k_t of inductions as function of time, respectively. Parameters: $\alpha = 0.05/\mu\text{m}^2$, $d_{\text{new}} = 10\ \mu$m, and $\eta = 1$. Fist induction occurs after 179 min.

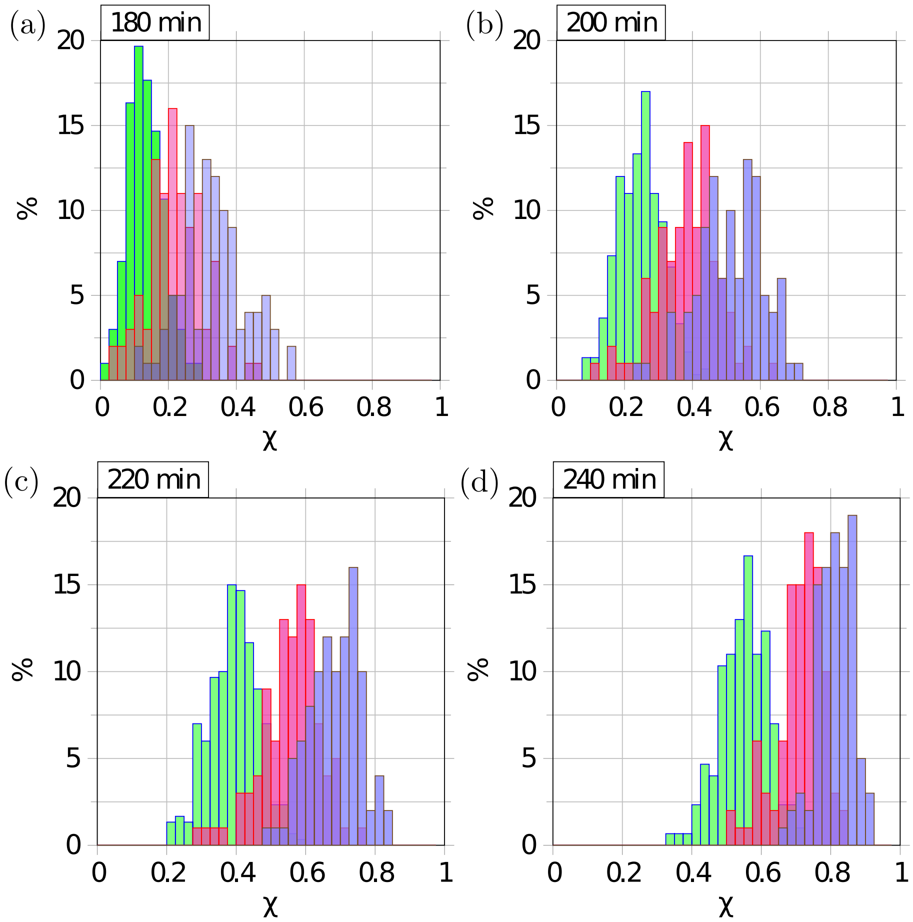

Figure S.4: The same as in figure 8 but for varying increase of AI production rate: $\eta = 1$ (blue), $\eta = 0.5$ (red), and $\eta = 0$ (green). Parameters: $\alpha = 0.25/\mu\text{m}^2$ and $\sigma_{\text{str}} = 20\ \mu\text{m}$.

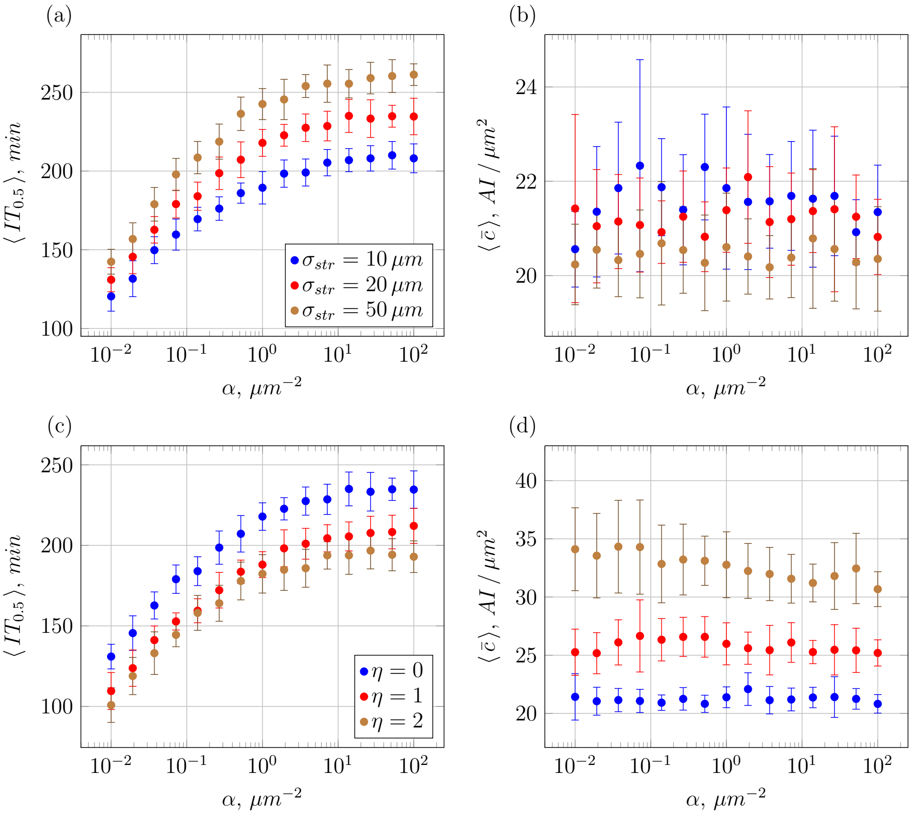

Figure S.5: Mean times when 50% of the cells of a population have been induced and corresponding mean concentrations as function of the inverse signal-range parameter α for different daughter cell spreads σ_{str} and rate of AI production increase η. Parameters: $\beta_{\mathrm{thr}} = 20$ AI/μm^2, $\eta = 0$ in (a) & (b) and $\sigma_{\mathrm{str}} = 20$ μm in (c) & (d).

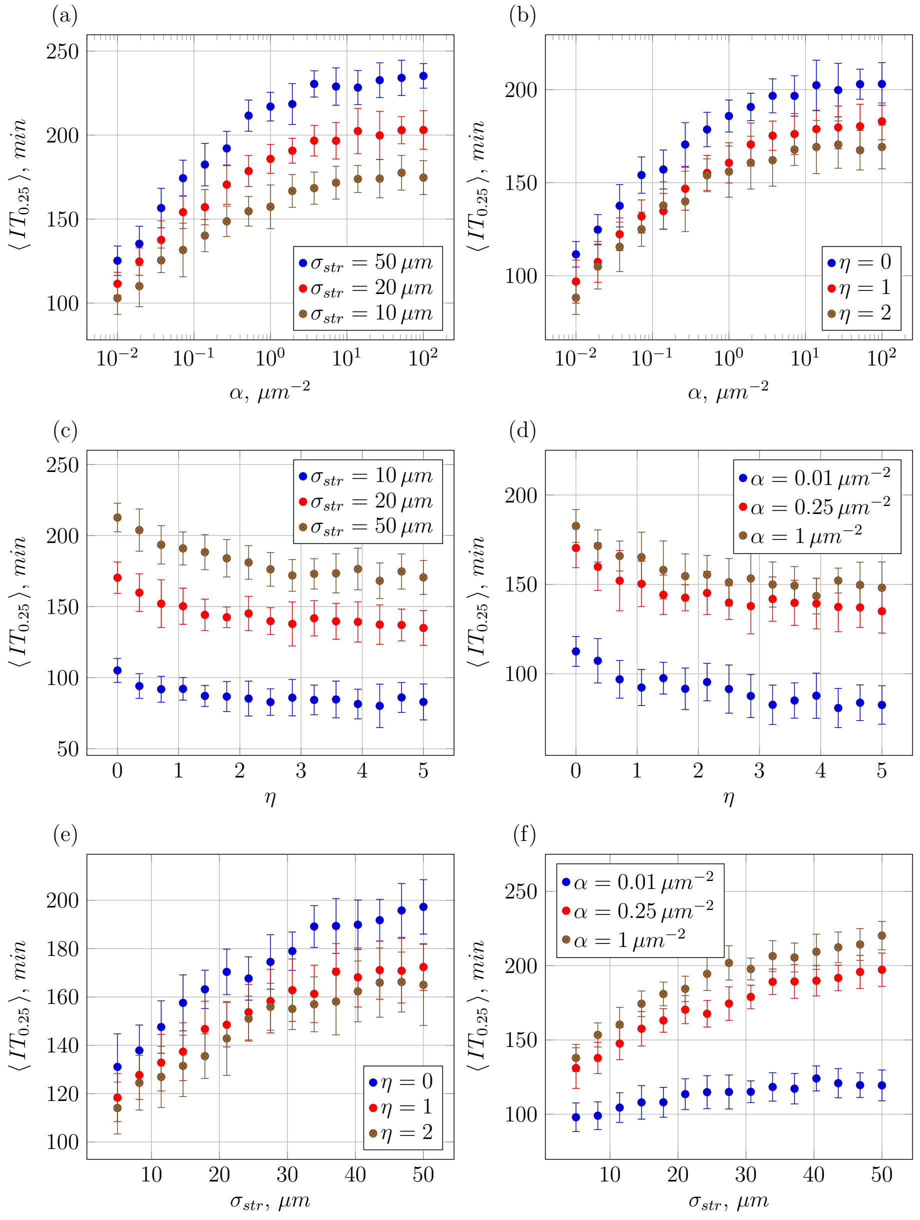

Figure S.6: Mean time when 25% of the population have been induced for different α, η, and $\sigma_{\rm str}$. Parameters: $\beta_{\rm str} = 20$ in units AI/μm^2 for all panels while $\alpha = 0.25/\mu\text{m}^2$ in panels (c) & (e), $\sigma_{\rm str} = 20\ \mu$m in (b) & (d), and $\eta = 0$ in (a) & (f).

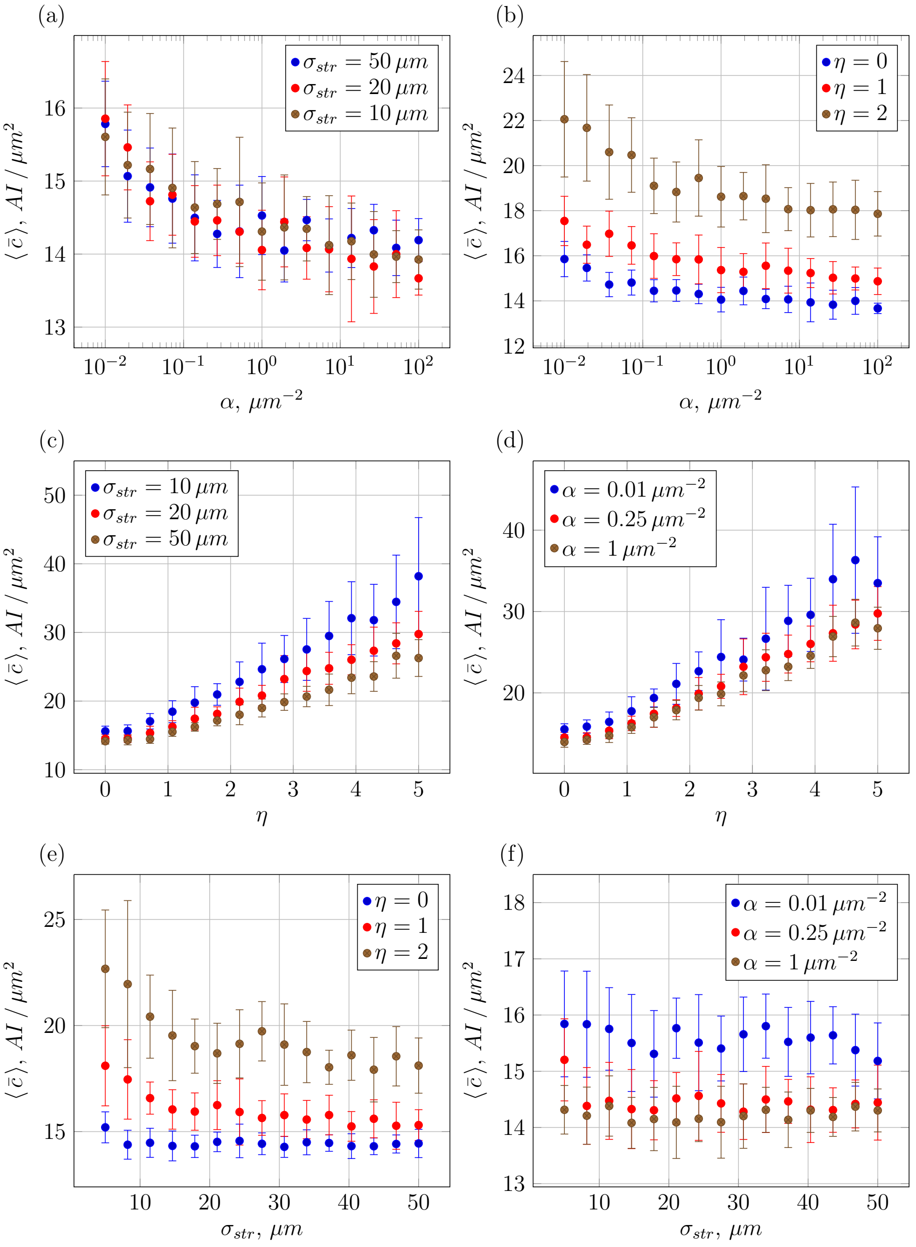

Figure S.7: Mean AI concentrations corresponding to figure S.6.

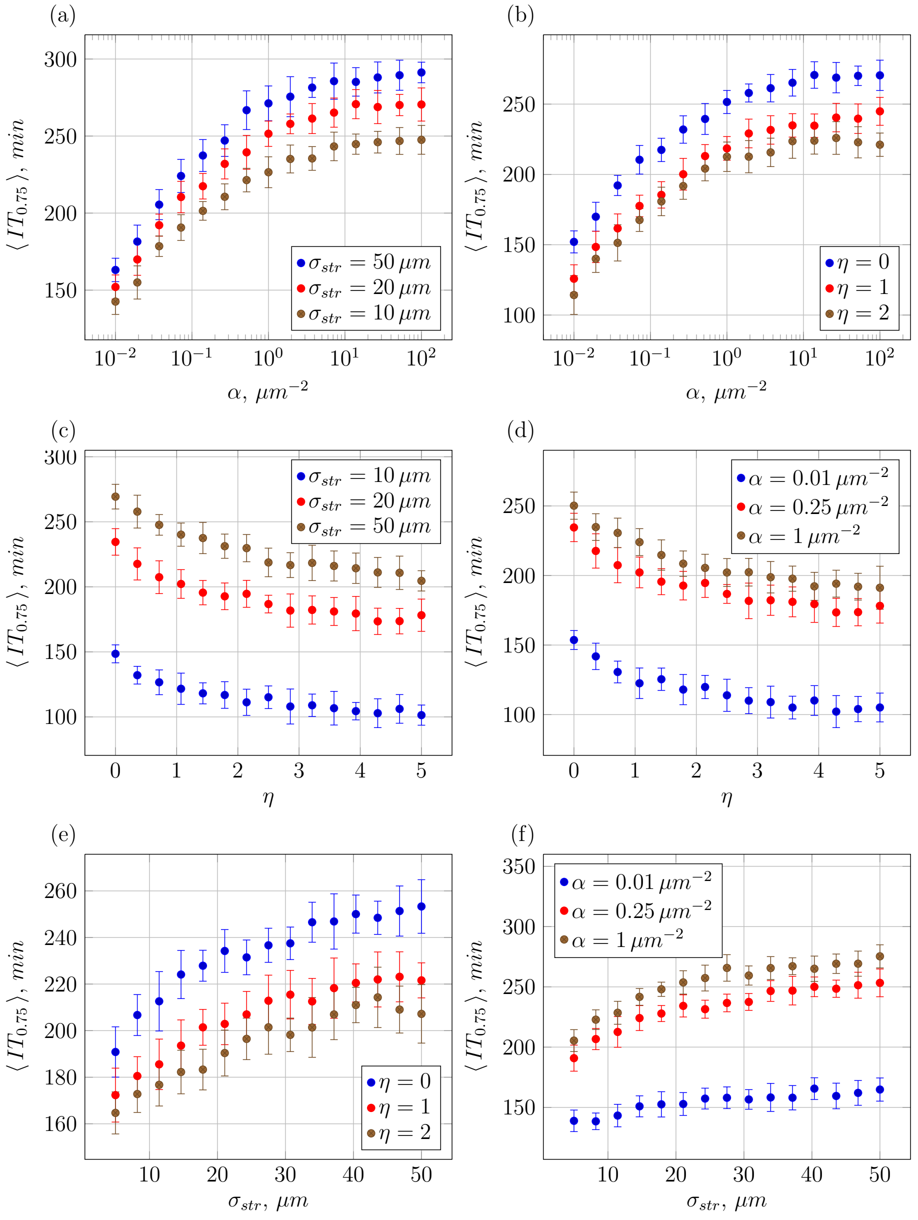

Figure S.8: Mean time when 75% of the population have been induced for different α, η, and σ_{str}. The parameters are the same as in figure S.6.

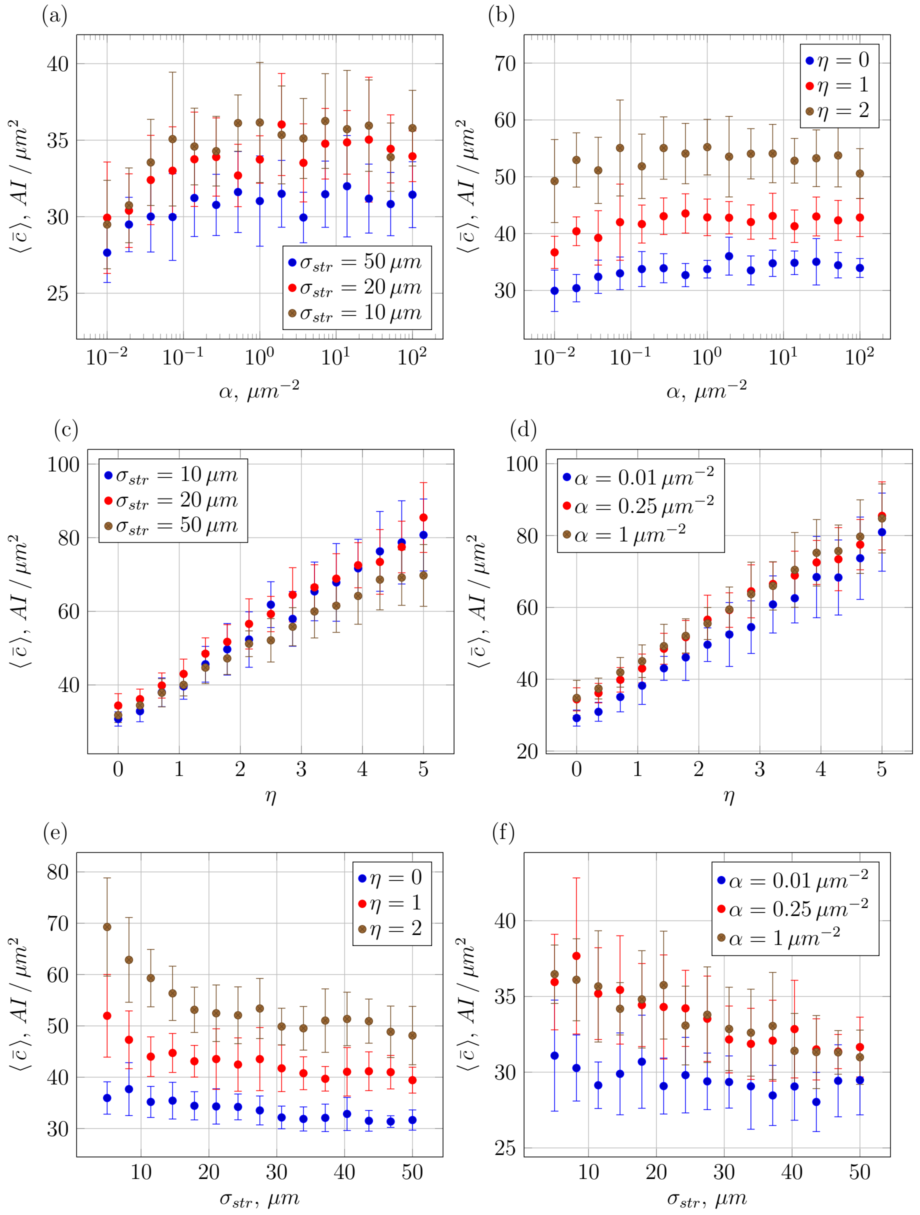

Figure S.9: Mean AI concentrations corresponding to figure S.8.

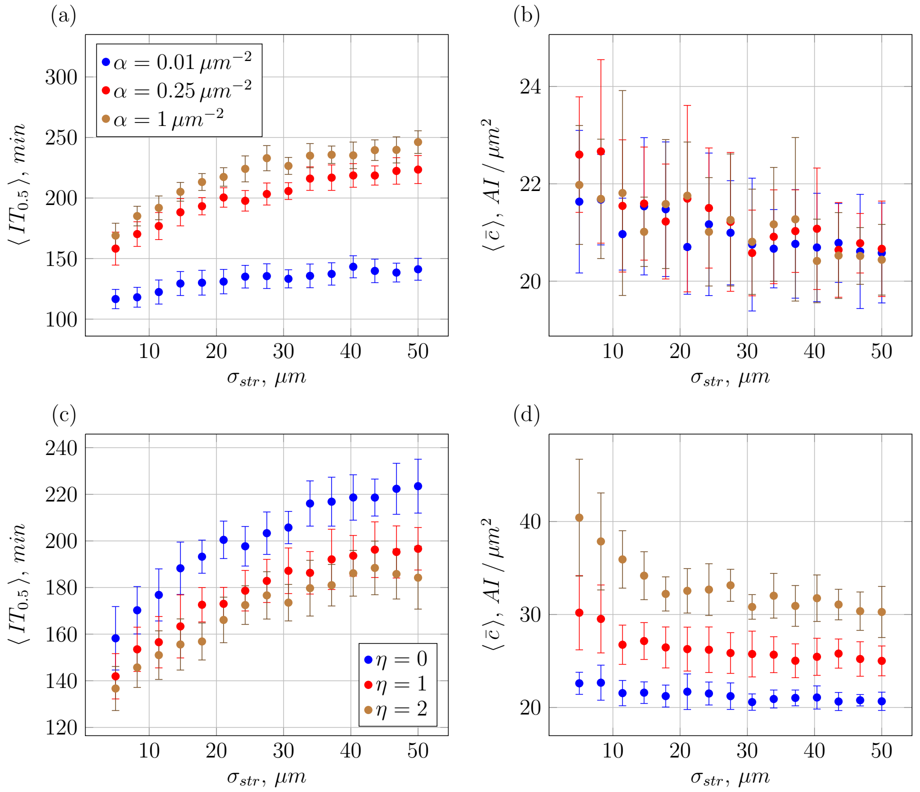

Figure S.10: Mean times when 50 % of the population have been induced and corresponding mean concentration as function of daughter displacement spread $\sigma_{\rm str}$ for different inverse signal-range parameters α and additional AI production rate η. Parameters: $\beta_{\rm str} = 20$ AI/μm^2, $\eta = 0$ in (c) & (d) and $\alpha = 0.25/\mu$m^2 in (a) & (b).

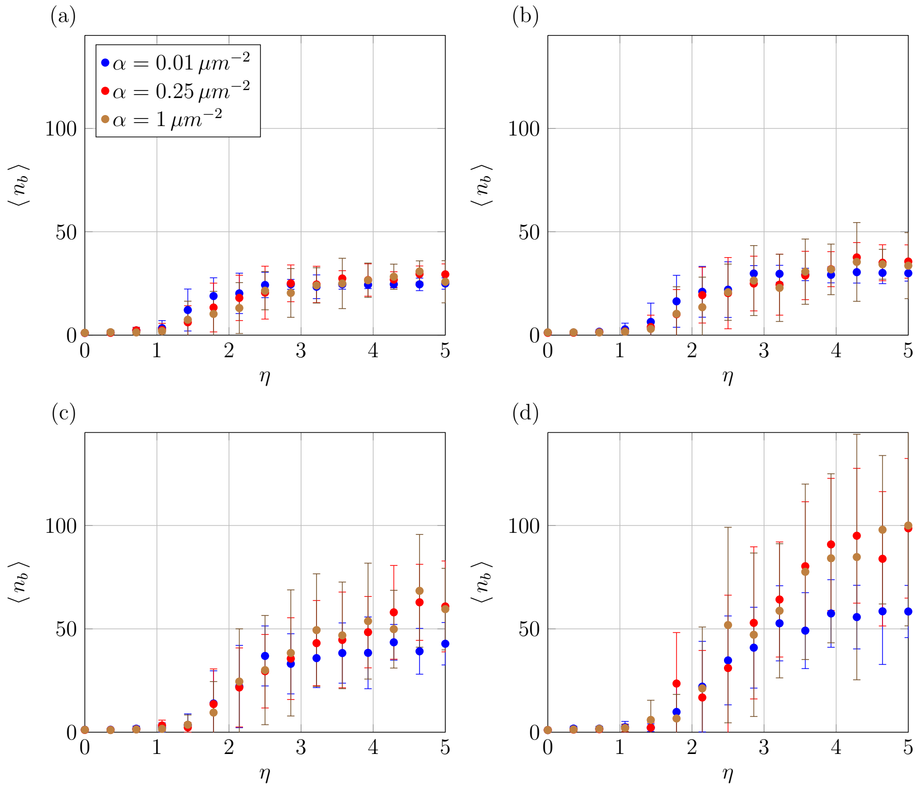

Figure S.11: Absolute mean burst size $\langle n_b \rangle$ corresponding to figure 12 as function of η for the same conditions.
